# Nitric oxide alleviates cell death through protein *S*-nitrosylation and transcriptional regulation during the ageing of elm seeds

**DOI:** 10.1093/jxb/ery270

**Published:** 2018-07-25

**Authors:** Yuqi He, Hua Xue, Ying Li, Xiaofeng Wang

**Affiliations:** 1 Beijing Advanced Innovation Center for Tree Breeding by Molecular Design, Beijing Forestry University, Haidian District, Beijing, PR China; 2 National Engineering Laboratory for Tree Breeding, College of Biological Sciences and Biotechnology, Beijing Forestry University, Haidian District, Beijing, PR China

**Keywords:** Cell death, metabolome, nitric oxide (NO), *S*-nitrosylation, seed ageing, seed deterioration, *Ulmus pumila* L

## Abstract

Seed ageing is a major problem in the conservation of germplasm resources. The involvement of possible signalling molecules during seed deterioration needs to be identified. In this study, we confirmed that nitric oxide (NO), a key signalling molecule in plants, plays a positive role in the resistance of elm seeds to deterioration. To explore which metabolic pathways were affected by NO, an untargeted metabolomic analysis was conducted, and 163 metabolites could respond to both NO and the ageing treatment. The primary altered pathways include glutathione, methionine, and carbohydrate metabolism. The genes involved in glutathione and methionine metabolism were up-regulated by NO at the transcriptional level. Using a biotin switch method, proteins with an NO-dependent post-translational modification were screened during seed deterioration, and 82 putative *S*-nitrosylated proteins were identified. Eleven of these proteins were involved in carbohydrate metabolism, and the activities of the three enzymes were regulated by NO. In combination, the results of the metabolomic and *S*-nitrosoproteomic studies demonstrated that NO could activate glycolysis and inhibit the pentose phosphate pathway. In summary, the combination of these results demonstrated that NO could modulate carbohydrate metabolism at the post-translational level and regulate glutathione and methionine metabolism at the transcriptional level. It provides initial insights into the regulatory mechanisms of NO in seed deterioration.

## Teaser

NO can modulate carbohydrate metabolism at the post-translational level and activate glutathione and methionine metabolism at the transcriptional level, alleviating cell death in elm seeds during ageing.

## Introduction

Seed ageing is an irreversible process of the gradual decline of seed vigour. It is characterized as a reduction of antioxidant systems, the disruption of cellular membranes, the damage of genetic integrity, the peroxidation of lipids, and the degradation of proteins in seeds. Recent studies found that the typical features of programmed cell death (PCD), such as DNA fragmentation, a TUNEL (terminal deoxynucleotidyl transferase dUTP nick end labelling)-positive nucleus, cytochrome *c* release, and an increase in caspase-3-like activity, were also present during seed deterioration (Hu *et al.*, 2012). A key modulator of PCD, reactive oxygen species (ROS), bursts early during the controlled deterioration treatment (CDT) and may induce downstream PCD events and dynamic changes in the mitochondria (Wang *et al.*, 2015). However, the involvement of other possible signal molecules in seed deterioration has not been described.

Nitric oxide (NO), a gaseous free radical, participates as a key messenger in multiple physiological processes in plants (Pagnussat *et al.*, 2002; Arc *et al.*, 2013; Yu *et al.*, 2014). NO could be produced in plants through a nitrite- or arginine-dependent pathway, and the latter is similar to the nitric oxide synthase (NOS)-dependent pathway present in animals (Astier *et al.*, 2018). NO could be generated during the early germination of seeds (Simontacchi *et al.*, 2004) and participate in promoting germination (Beligni and Lamattina, 2000) and breaking dormancy (Bethke *et al.*, 2006a, b). Further study found that the NO-induced *S*-nitrosylation modification of ABI5 facilitates its degradation and promotes seed germination (Albertos *et al.*, 2015). Several studies have focused on the roles of NO in plant senescence and cell death. For example, senescent pea leaves produced less NO than young leaves in vascular tissues, indicating that NO could be involved in the process of leaf senescence (Corpas *et al.*, 2004). In the Arabidopsis mutant *noa1* with a defective *nitric oxide associated 1* gene, the detached leaves and intact plants senesced more rapidly than the wild type, and treatment with the NO donor sodium nitroprusside (SNP) slowed the dark-induced senescence of the *noa1* mutant leaves (Guo and Crawford, 2005). The overexpression of an NO-degrading dioxygenase (NOD) in Arabidopsis initiates a senescence-like phenotype (Mishina *et al.*, 2007). The leaves from *dnd1*, a plasma membrane-localized cation channel mutant, contained less NO than the wild-type leaves. They senesced faster than the wild-type leaves, and the application of SNP rescued the senescence-related phenotypes (Ma *et al.*, 2010). These studies provide evidence that NO plays an important role in delaying leaf senescence and cell death. However, its exact role in seed deterioration has not been investigated.

NO could regulate plant cell death by modulating related gene expression. For example, when SNP was used to induce hypersensitive cell death, it activated the expression of the glutathione *S*-transferase (GST) and glutathione peroxidase (GPX) genes in Arabidopsis leaves, suggesting that NO is related to ROS generation and removal (Polverari *et al.*, 2003). Zago *et al.* (2006) found that the expression of *S*-adenosyl-l-methionine synthetase (SAMS), which catalyses the conversion of l-methionine into the ethylene precursor *S*-adenosyl-l-methionine (AdoMet), was increased when SNP was used to treat tobacco leaves during moderately high light-induced cell death. In Arabidopsis leaves, SNP attenuated the activation of the genes for salicylic acid (SA) biosynthesis in O_3_-induced cell death (Ahlfors *et al.*, 2009). These studies illustrated that NO regulates cell death by modulating gene expression and inducing metabolic changes.

In recent years, an NO-dependent post-translational modification, protein *S*-nitrosylation, was found widely in plants (Besson-Bard *et al.*, 2008). *S*-Nitrosoglutathione (GSNO) was a potential NO pool that regulates protein function by *S*-nitrosylation (Corpas *et al.*, 2013), and *S*-nitrosoglutathione reductase (GSNOR) was responsible for the removal of GSNO (Begara-Morales *et al.*, 2016). The protein level and activity of GSNOR were down-regulated during pepper fruit ripening, while the level of global *S*-nitrosylation was increased (Rodríguezruiz *et al.*, 2016). The activity and stability of GSNOR were modulated by its *S*-nitrosylation (Guerra *et al.*, 2016; Tichá *et al.*, 2017). Global *S*-nitrosylation was involved in the regulation of plant cell death. The Arabidopsis *GSNOR1* mutant has a higher NO level (Chen *et al.*, 2009), increased protein *S*-nitrosylation (Hu *et al.*, 2015), and an anti-cell death phenotype (Chen *et al.*, 2009). Subsequently, de Pinto *et al.* (2013) found that both GSNO and proteic-SNO groups quickly increased in the H_2_O_2_-induced PCD of tobacco Bright Yellow-2 cells, while the enzymatic activity and gene expression of GSNOR decreased, providing evidence that global *S*-nitrosylation was involved in H_2_O_2_-induced PCD. In addition, it was reported that the *S*-nitrosylation of individual proteins played important roles in cell death. Glyceraldehyde-3-phosphate dehydrogenase (GAPDH) and thioredoxin (TRX), previously reported to be involved in cell death in animals, were identified as being *S*-nitrosylated in the *noe1* mutant of rice undergoing leaf cell death, implying that an *S*-nitrosylation-related model similar to animal cell death might exist during plant cell death (Lin *et al.*, 2012). In addition, the level of ROS and the antioxidant systems was also found to be controlled by the *S*-nitrosylation of some proteins. For example, the *S*-nitrosylation of the NADPH oxidase AtRBOHD at Cys890 abolished its ability to synthesize reactive oxygen intermediates, resulting in a perturbation of the magnitude of cell death development (Yun *et al.*, 2011). Enzymes involved in the ascorbate–glutathione cycle, including ascorbate peroxidase (Begara-Morales *et al.*, 2014; de Pinto *et al.*, 2013), monodehydro-ascorbate reductase, and dehydro-ascorbate reductase, were also found to be regulated by *S*-nitrosylation (Begara-Morales *et al.*, 2016). These functions of *S*-nitrosylation prompted us to study how this post-translational modification works in cell death during seed deterioration.

Global protein *S*-nitrosylation was detected during plant cell death (Lin *et al.*, 2012) under numerous types of stress (Tanou *et al.*, 2012), which was usually labelled with biotin and enriched by affinity chromatography. Ultra-performance liquid chromatography quadrupole-time of fight mass spectrometry (UPLC-Q-TOF MS) is a technique with high resolution and high throughput used in metabolomics analyses. Not only can it detect primary metabolites, but it can also detect secondary metabolites that are especially rich in plants (De Vos *et al.*, 2007). In this study, the combination of *S*-nitrosylated proteome and metabolite analyses were adopted to explore the overall role and mechanisms of NO in seed deterioration. We initially found that NO plays an active role in the resistance of elm seed to deterioration and explored the versatile effects of NO on metabolic contents, gene expression, and protein *S*-nitrosylation. These results combined to provide initial insights into the regulatory role of NO on carbohydrate, glutathione, and methionine metabolism in cell death during seed deterioration.

## Materials and methods

### Materials

Elm seeds (*Ulmus pumila* L.) were obtained from plants grown at the Beijing Forestry University, China. The original germination percentage was 98%, and the moisture content was 0.077 g H_2_O g^−1^ DW. Germination assays were performed as previously described (Hu *et al.*, 2012). The seeds were stored at –20 °C in tightly closed containers until required for analysis.

### Controlled deterioration treatment (CDT) and pharmacological treatments

CDT was performed as previously described (Hu *et al.*, 2012). Seeds equilibrated in sealed bottles at 37 °C and 100% relative humidity for 1 d are defined as post-equilibration (PE). Before the germination tests, SNP, GSNO, reduced glutathione (GSH), and 2-(4-carboxyphenyl)-4,4,5,5-tetramethylimidazoline-1-oxyl-3-oxide (c-PTIO) purchased from Sigma-Aldrich were used for pre-treatment. The SNP treatment was performed as described by Bethke *et al.* (2006*a*) with some modifications. Seeds were exposed to vapours from different concentrations of SNP or H_2_O for 12 h and re-dried to their original moisture content and aged as described by Wang *et al.* (2015). For other treatments, the seeds were pre-treated with GSNO (100 μM), GSH (250 μM), c-PTIO (50 μM), or H_2_O for 12 h at 4 °C and re-dried and aged. The vigour index was calculated using the following formula: VI=*G*_*t*_×*S*_*x*_ where *G*_*t*_ represents germination at time *t*, and *S*_*x*_ represents the average radicle weight after germination.

### Triphenyltetrazolium chloride (TTC) staining

The two pieces of cotyledon from the elm seeds were incubated with distilled water for 12 h, placed on two layers of filter paper, and soaked in 0.25% (w/v) TTC solution for 12 h in the dark at room temperature.

### Measurement of NO levels

The visualization of NO was performed with the specific NO fluorescent probe DAF-FM DA (Sigma-Aldrich) using the method described by Kojima *et al.* (1999) with slight modifications. The testa of the seeds was removed, and the seeds were cut along the axis with an oscillating slicer. The sections were incubated in loading buffer (PBS, pH 7.4) with DAF-FM DA at a final concentration of 10 μM for 1 h in the dark at 25 °C and rinsed with loading buffer three times for 15 min. All the images were visualized using laser scanning confocal microscopy (LSCM, excitation at 488 nm and emission at 515 nm). The experiment was repeated three times. The signal intensities of the green fluorescence in the images of the seeds were quantified as described by Wilkins *et al.* (2011) by measuring the average pixel intensity using ImageJ software. The NO level in the whole seeds was also measured using a spectrofluorometer. Fluorescence was measured with a 485 nm excitation and a 535 nm emission filter for 12 h (Rasul *et al.*, 2012).

### Untargeted metabolomic analysis

Untargeted metabolomic analysis was performed using UPLC-Q-TOF MS. For each sample, 0.1 g of seeds were frozen in liquid nitrogen and lyophilized. Three biological replicates were analysed for each sample. The metabolomics procedures were conducted as previously described (Zhang *et al.*, 2014).

Principal component analysis (PCA) was performed for the unsupervised multivariate statistical analysis. Orthogonal projection to latent structure discriminant analysis (OPLS-DA) was performed as a supervised method to isolate the metabolites responsible for the differences among the six treatments. OPLS-DA models were validated based on the multiple correlation coefficient (*R*^2^) and cross-validated *R*^2^ (*Q*^2^) in cross-validation and permutation tests by applying 2000 iterations (*P*>0.001). The significance of the biomarkers was ranked using the variable importance in projection (VIP) score (>1).

### Total RNA extraction, cDNA synthesis and real-time quantitative PCR (qRT-PCR) analysis

Total RNA extraction, cDNA synthesis, and qRT-PCR were conducted as described by Peng *et al.* (2017). All the primers used are listed in Supplementary Table S1 at *JXB* online.

### Determination of oxidized glutathione (GSSG) and reduced glutathione (GSH) content

GSSG and GSH contents were determined as described by Griffith *et al.* (1980) using the 5,5'-dithiobis-(2-nitrobenzoic acid)-GR recycling procedure.

### Determination of *S*-nitrosothiol (SNO) content and the identification of *S*-nitrosylated proteins

The SNO content was quantified using the Saville–Griess assay as previously described (Saville, 1958). The identification of the *S*-nitrosylated proteins was performed as previously described (Forrester *et al.*, 2009) with some modifications. The seeds were ground in liquid nitrogen and homogenized in HEN buffer (25 mM HEPES-NaOH, pH 7.7, 50 mM NaCl, 0.1 mM EDTA, and 0.1 mM neocuproine). The supernatant was then blocked with 20 mM methyl methanethiosulphonate and 2.5% SDS in HEN buffer. After acetone precipitation, the pellet was re-suspended in HEN buffer containing 1% SDS. The proteins were labelled with 2 mM biotin-HPDP, and the reaction was initiated by 200 mM sodium ascorbate. Proteins untreated with biotin-HPDP and sodium ascorbate were used as a control. Finally, the pellet was analysed using either immunoblotting with anti-biotin antibody (Cayman) or affinity purification using streptavidin magnetic beads (NEB). The pellet was re-suspended in neutralization buffer (25 mM HEPES-NaOH, 100 mM NaCl, 1 mM EDTA, and 0.5% Triton X-100, pH 7.5) and transferred to bind with streptavidin beads. The beads were washed four times with neutralization buffer containing 600 mM NaCl and eluted with HEN buffer containing 1% β-mercaptoethanol. Protein digestion was performed as described by Wiśniewski *et al.* (2009). The proteins were identified using HPLC-MS/MS as described by Zhang *et al.* (2015). Protein functional classification was conducted using the program Gene Ontology (GO) with respect to its cellular component, biological process, and molecular function using GO annotation or annotated manually based on literature searches and closely related homologues (Zhang *et al.*, 2015).

### Enzyme activity assays

For the *in vivo* experiments, the seeds were pre-treated with SNP (100 μM), GSNO (100 μM), and c-PTIO (50 μM) for 12 h at 4 °C and re-dried and aged for 3 d. The seeds were homogenized in the HEN buffer, and the homogenate was centrifuged at 13000 *g* for 10 min at 4 °C. For the *in vitro* treatment, the seeds of different CDTs were homogenized, and the supernatant was pre-incubated with or without GSNO (100 μM), GSH (250 μM), or DTT (10 mM) in the dark for 30 min at 25 °C. The protein concentrations of the seed extracts were determined using the Bradford method. For each enzymatic assay, three extractions were conducted as replicates.

6-Phosphogluconate dehydrogenase (6PGDH; EC 1.1.1.44) activity was determined using a spectrophotometer to detect the 6-phosphogluconate-dependent reduction of NADP^+^ at 340 nm (Leterrier *et al.*, 2012). Pyrophosphate-fructose 6-phosphate 1-phosphotransferase (PFP; EC 2.7.1.90) activity was determined based on the disappearance of NADH accompanying the formation of fructose 1,6-bisphosphate (F-1,6-P_2_) in the presence of fructose bisphosphate aldolase (FBA; EC 4.1.2.13), triose phosphate isomerase (TPI, EC 5.3.1.1), and GAPDH (EC 1.1.1.8) (Mustroph *et al.*, 2013). Phosphoglucomutase (PGM; EC 5.4.2.2) activity was measured using an enzyme-coupled assay containing glucose 6-phosphate dehydrogenase (G6PDH) to convert glucose-1-phosphate to 6-phosphoglyceraldehyde as described by Hattenbach and Heineke (1999). The FBA activity was measured based on Boyer’s modification of the hydrazine assay (Sehrawat *et al.*, 2013*a*). TPI activity was measured by converting dihydroxyacetone phosphate (DHAP) to glyceraldehyde 3-phosphate (G3P) coupled with GAPDH as described by Pichersky and Gottlieb (1984). Malate dehydrogenase (MDH; EC 1.1.1.37) activity was determined by monitoring the increase in absorbance at 340 nm due to NADH production (Xin *et al.*, 2014). Transketolase (TKL; EC 2.2.1.1) activity was determined using an enzyme-linked method (Haake *et al.*, 1998).

### Statistical analysis

Univariate statistics were performed using a one-way ANOVA with Tukey’s post-tests using SPSS (version 19, SPSS Inc., Chicago, IL, USA). Significant analyses were performed using Duncan’s multiple range test at *P*<0.05. The values are presented as the means ±SE.

## Results

### NO enhanced the resistance of seeds to ageing

**Fig. 1.**
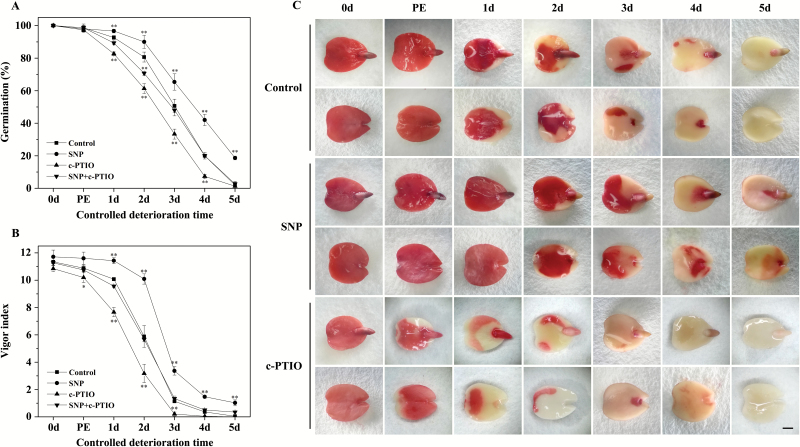
Effect of NO on seed vigour during controlled deterioration treatment (CDT). (A, B) Effects of SNP and c-PTIO on the germination percentage (A) and the vigour index (B) of ageing seeds. Seeds were treated with either 100 μM SNP vapour or 50 μM c-PTIO, or imbibed with c-PTIO and then treated with SNP vapour, and the percentage of germination was calculated after 7 d of imbibition. Data are presented as the mean ±SD of three replicates. PE, post-equilibration. *P<0.05; **P<0.01. (C) Effect of SNP (100 μM) or c-PTIO (50 μM) on the TTC staining of seeds during CDT. Light micrographs showed the two pieces of elm cotyledon after the TTC staining. PE, post-equilibration. Scale bars=1 mm.

Since NO seems to be a key participant in the regulation of plant senescence (Mishina *et al.*, 2007), we first investigated the possible involvement of NO in the CDT of elm seeds. When the elm seeds were exposed to vapours from an NO donor SNP, the germination percentage of the ageing seeds was affected (Fig. 1A). The germination percentage was enhanced with a moderate concentration of SNP (100 μM) from 51% to 65% after 3 d of CDT, and it even increased from 2% to 18% after 5 d of CDT. However, lower (50 μM) or higher (150 μM) concentrations of SNP had no effect on the vigour of the aged seeds, showing a narrow window of SNP concentration in the resistance to seed ageing (Supplementary Fig. S1). To confirm further the role of NO in seed ageing, elm seeds pre-treated with the NO scavenger c-PTIO and/or SNP were subjected to CDT, after which the seed vigor was determined (Fig. 1B). Pre-treatment with 100 μM SNP delayed seed ageing, while imbibition with 50 μM c-PTIO accelerated the loss of seed vigour, and the effect was significant at each time point. Co-imbibition with c-PTIO could counteract the effect of SNP during CDT. These data suggested the possible involvement of NO in the regulation of seed vigour during CDT.

Seed viability was further evaluated using the TTC reduction assay. The non-aged seeds were stained deep red throughout, and the stained part shrank with prolonged ageing, which demonstrated that the seed vigour was gradually decreasing during CDT (Fig. 1C). SNP delayed the fading of the red stain, while c-PTIO accelerated this process. These results, together with the germination data, demonstrated that NO can reduce cell death during the ageing of elm seeds, and cell viability is lost earlier than the germination capacity.

### NO production during CDT-induced seed ageing

**Fig. 2.**
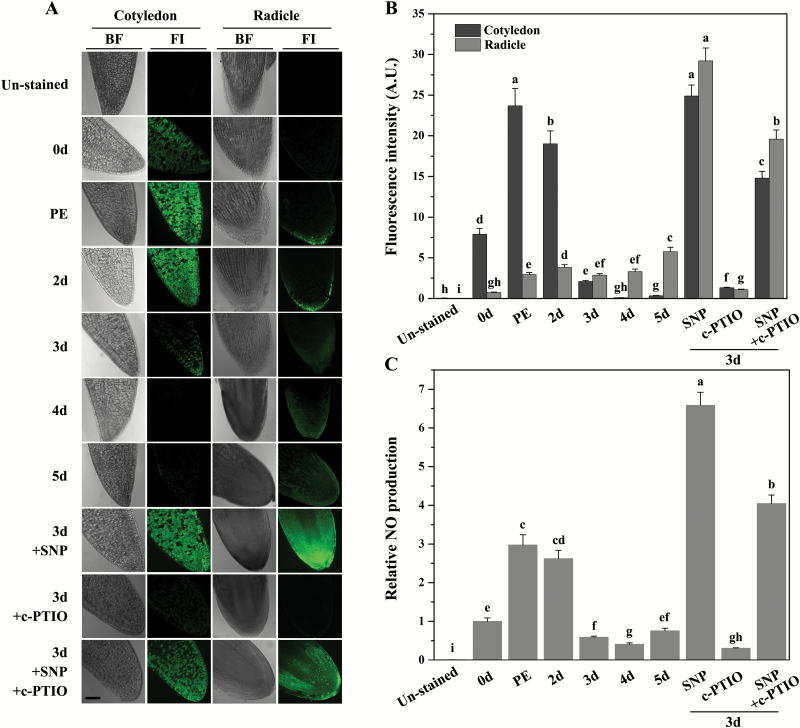
NO production during seed deterioration. The seeds were subjected to CDT for 0 d, post-equilibration (PE), 2, 3, 4, and 5 d, or pre-treated with 100 μM SNP, 50 μM c-PTIO, or SNP plus c-PTIO before 3 d of CDT. (A) Representative fluorescent images of NO production in ageing seeds. Seed sections were incubated with DAF-FM DA and detected using confocal laser scanning microscopy. The cotyledon and radicle were observed separately. Bright field (BF) and fluorescence images (FI) are listed in different columns. Scale bars=0.1 mm. (B) Relative DAF-FM DA fluorescence signal densities of (A). Data are the mean ±SD of six replicates. Different superscript letters indicate a significant difference of the fluorescence signal at *P*<0.05. (C) Spectrofluorometric analysis of NO levels in whole seeds during ageing. Data are the mean ±SD of six replicates. Different superscript letters indicate a significant difference in the fluorescence signal at *P*<0.05.

Based on the effects of SNP and c-PTIO on seed ageing, the production of NO during ageing was detected using the fluorescent probe DAF-FM DA, and the signal intensities were quantified (Fig. 2A, B). In non-aged seeds, there is a visible NO signal in the cotyledons but not in the radicles. At an early stage of ageing, the NO levels dramatically increased and remained at a higher level in cotyledons after 2 d of CDT. However, the signals rapidly decreased at day 3 and almost disappeared after 4 d of CDT. In the radicles, the NO signal was visualized in the cap at PE and day 2 of CDT, and then gradually spread inside. Spectrofluorometric analysis of the NO level in the whole seeds also demonstrated an endogenous NO burst at the early stages of ageing that later dropped to a lower level (Fig. 2C). The spatial specificity of the NO distribution may be associated with different cell viability in the cotyledon and radicle (i.e. the cotyledon contained less NO at the late stage of ageing and its vigour was lost earlier than that of the radicle) as illustrated by TTC staining (Fig. 1C).

To confirm the reliability of the NO fluorescence data, seeds aged for 3 d were pre-treated with SNP alone, c-PTIO alone, or both agents combined (Fig. 2A–C). SNP treatment greatly increased the green fluorescence throughout the cell, and c-PTIO diminished the signal to half that of the untreated control, confirming the specificity of the NO production induced by CDT. When the seeds were pre-treated with both SNP and c-PTIO, the SNP-induced fluorescence burst was counteracted by c-PTIO. These results supported a hypothesis that the vigour suppression effects of c-PTIO on aged seeds were attributed to a decrease in NO levels. Taken together, it was demonstrated that NO played a positive role in the resistance to seed deterioration.

### Detection and identification of differential metabolites

**Fig. 3.**
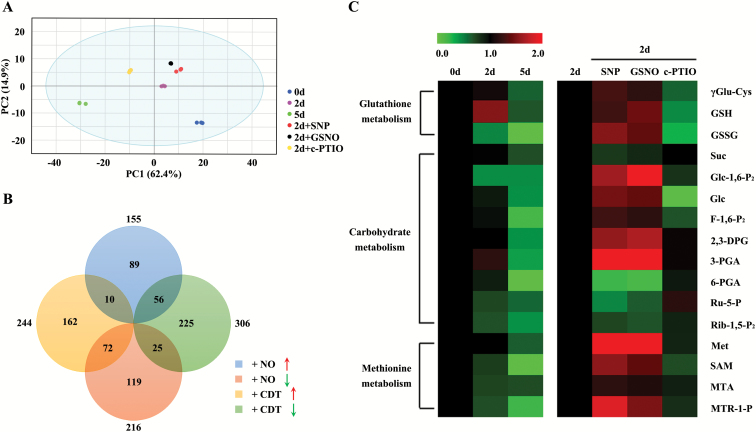
PCA plots and heat map of the metabolites analysed by UPLC-Q-TOF MS in six treatments. Seeds were subjected to CDT for 0, 2, and 5 d, or pre-treated with 100 μM SNP, 100 μM GSNO, or 50 μM c-PTIO and then subjected to CDT for 2 d. (A) Unsupervised PCA score plots derived from the UPLC-Q-TOF MS data. (B) Comparison of metabolites changes in the CDT and NO treatment. (C) Heat map of the metabolic changes in glutathione metabolism, carbohydrate metabolism, and methionine metabolism in response to CDT or NO. γGlu-Cys, γ-Glutamyl-cysteine; GSH, glutathione; GSSG, oxidized glutathione; Suc, sucrose; Glc-1,6-P_2_, α-d-glucose 1,6-bisphosphate; Glc, d-glucose; F-1,6-P_2_, fructose 1,6-bisphosphate; 2,3-DPG, 2,3-diphosphoglyceric acid; 3-PGA, 3-phosphoglyceric acid; 6-PGA, 6-phosphogluconic acid; Ru-5-P, d-ribulose 5-phosphate; Rib-1,5-P_2_, ribose 1,5-bisphosphate; Met, l-methionine; SAM, *S*-adenosylmethionine; MTA, 5-methylthioadenosine; MTR-1-P, 5-methylthioribose 1-phosphate.

To analyse the effects of NO on seed metabolism during CDT, a non-targeted metabolomics study was performed. A total of 10472 molecular features were extracted from samples of non-aged seeds, seeds subjected to CDT for 2 d and 5 d, and seeds pre-treated with SNP, GSNO, or c-PTIO before CDT for 2 d using Progenesis QI software. The results of the PCA showed that the metabolomics of the six groups were clearly separated by PC1 (62.4%) and further separated by PC2 (14.9%) (Fig. 3A). The reliable data quality across all the samples demonstrated the suitability of the method for metabolic profiling studies during the experiment. To identify the metabolites significantly affected by CDT or the reagent treatments, OPLS-DA modelling was performed, and a clear separation of CDT for 2 d (*R*^2^=0.98, *Q*^2^=0.999)/5 d (*R*^2^=0.997, *Q*^2^=0.999) from the non-aged sample and SNP (*R*^2^=0.966, *Q*^2^=0.987)/GSNO (*R*^2^=0.985, *Q*^2^=0.991)/c-PTIO (*R*^2^=0.991, *Q*^2^=0.999) pre-treated seeds from the non-treated sample (Supplementary Fig. S2) was observed. A total of 550 significantly changed metabolites were selected to be potential biomarkers related to CDT based on the VIP score (>1). Among them, 244 metabolites significantly increased and 306 decreased after CDT (Fig. 3B).

The concentration of 371 metabolites significantly changed after treatment with NO, and 155 metabolites increased and 216 decreased significantly. Unexpectedly, a combined analysis showed that 163 metabolites could respond to both the NO and ageing treatments (Fig. 3B). These overlaps illustrated that NO may participate in the regulation of seed deterioration. Supplementary Table S2 lists metabolites identified (compound name, molecular formula, and adduct) and their corresponding fold changes in concentration.

To identify possible pathways that are affected by NO, the metabolites were searched against the KEGG pathway database (http://www.genome.jp/kegg/) for their corresponding metabolic pathways. In this manner, three different metabolic pathways, namely glutathione, carbohydrate, and methionine metabolism, were found to be affected by NO during seed deterioration (Fig. 3C). The results showed that three metabolites involved in glutathione metabolism and four metabolites in methionine metabolism significantly increased with the SNP or GSNO treatments and decreased after c-PTIO treatment, suggesting that they were up-regulated by NO. Nine metabolites involved in carbohydrate metabolism exhibited a complicated change after the NO treatments. Five metabolites involved in overall glycolysis increased with the SNP or GSNO treatments and decreased after c-PTIO treatment. Three metabolites involved in the pentose phosphate pathway (PPP) were down-regulated after the SNP or GSNO treatments. These data indicated that NO could activate glycolysis and inhibit the PPP. Almost all the metabolites in these three pathways decreased during ageing, with the exception of GSH and 3-phosphoglyceric acid (3-PGA) that increased at 2 d of CDT and then decreased.

### qRT-PCR analysis for the genes involved in specific metabolic pathways

**Fig. 4.**
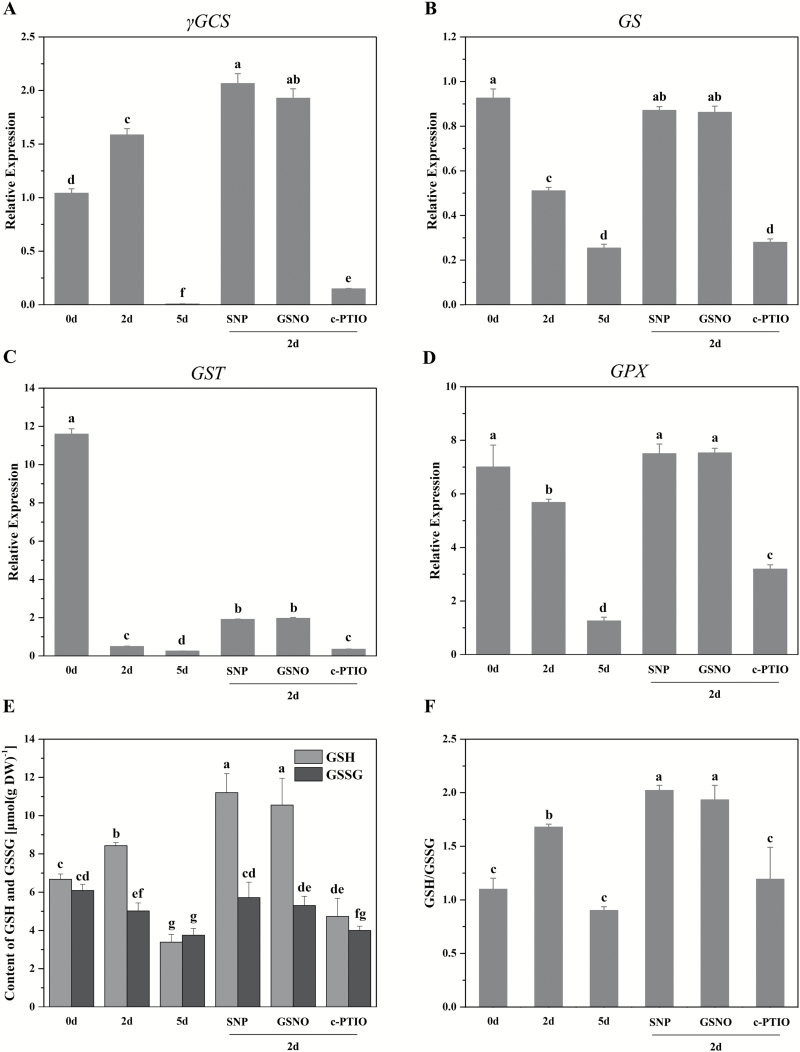
Effects of NO on glutathione metabolism. (A–D) The expression pattern of genes in glutathione metabolism subjected to different treatments using qRT-PCR. *P*<0.05. *γGCS*, γ-glutamylcysteine synthetase; *GS*, glutathione synthase; *GST*, glutathione S-transferase; *GPX*, glutathione peroxidase. (E, F) The effects of NO on the content of GSH and GSSG (E), and the ratio of GSH to GSSG (F). *P*<0.05. GSH, reduced glutathione; GSSG, oxidized glutathione.

To explore the molecular mechanism of metabolic changes in response to NO during CDT, 19 genes putatively participating in glutathione, methionine, and carbohydrate metabolism were analysed using qRT-PCR. As shown in Fig. 4, the treatments with SNP or GSNO significantly increased the expression of γ-glutamylcysteine synthetase (γGCS; Fig. 4A), glutathione synthase (GS; Fig. 4B), GST (Fig. 4C), and GPX (Fig. 4D), and c-PTIO could decrease their expression. The expression level of γGCS, the rate-limiting enzyme of glutathione metabolism, increased significantly after 2 d of CDT and then decreased sharply. This could explain the transient increase in GSH at the early stage of ageing (Fig. 4E). The expression level of the other enzymes, GS, GST, and GPX, declined continuously during ageing. Since GSH/GSSG was the most widely used indicator for the redox state of the GSH pool, we measured the contents of GSH and GSSG (Fig. 4E, F), and found that their responses to NO and CDT were consistent with the results obtained in the metabolomics analysis (Fig. 3C). In addition, the ratio of GSH to GSSG increased after SNP or GSNO treatment, while it decreased after c-PTIO treatment. These results illustrated that NO induced the expression of γGCS, GS, GST, and GPX, leading to an increase in GSH/GSSG, and protected the seeds from oxidative damage during deterioration.

**Fig. 5.**
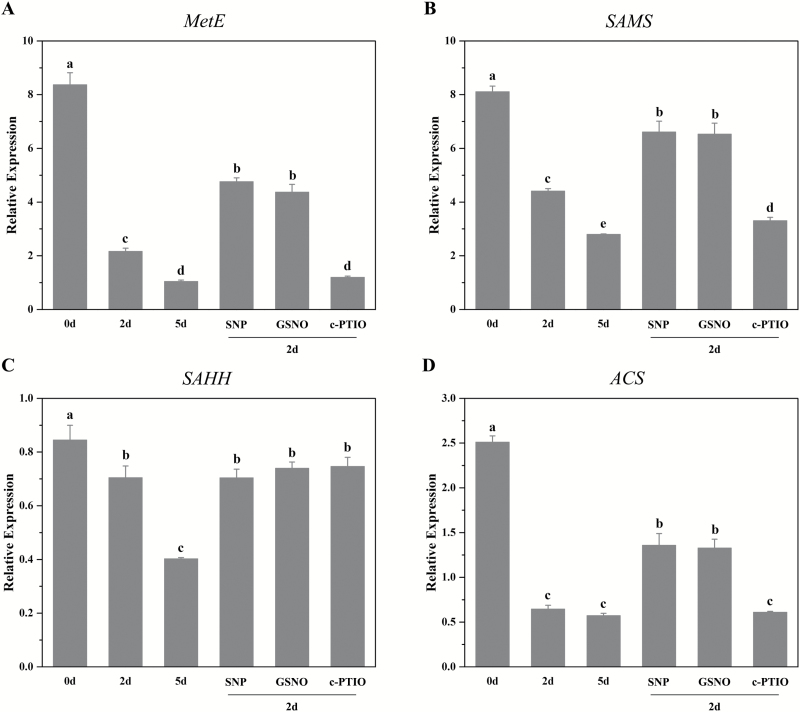
Effects of NO on gene expression in methionine metabolism. (A–D) The expression pattern of genes in methionine metabolism with different treatments using qRT-PCR. *P*<0.05. *MetE*, 5-methyltetrahydropteroyltriglutamate-homocysteine *S*-methyltransferase; *SAMS*, *S*-adenosyl-l-methionine synthetase; *SAHH*, *S*-adenosyl homocysteinase; *ACS*, 1-aminocyclopropane-1-carboxylic acid synthase.

During ageing, the expression level of 5-methyltetrahydropteroyltriglutamate-homocysteine *S*-methyltransferase (*MetE*; Fig. 5A), *SAMS* (Fig. 5B), *S*-adenosyl homocysteinase (*SAHH*; Fig. 5C), and 1-aminocyclopropane-1-carboxylic acid synthase (*ACS*; Fig. 5D) in methionine metabolism continuously declined. Pre-treatments with SNP or GSNO significantly increased the expression of *MetE*, *SAMS*, and *ACS*, and c-PTIO decreased their expression. The transcription of *SAHH*, encoding an enzyme in bypass of methionine metabolism, was not affected by these treatments. These results were consistent with the changes in the metabolites and illustrated that NO could activate methionine metabolism by improving the transcription of the *MetE*, *SAMS*, and *ACS* genes. It is worth noting that the genes involved in carbohydrate metabolism generally decreased during seed deterioration, but were not significantly altered after NO treatment (Supplementary Fig. S3), which illustrated that NO might not modulate carbohydrate metabolism at the transcriptional level.

### Protein *S*-nitrosylation during CDT-induced seed ageing

**Fig. 6.**
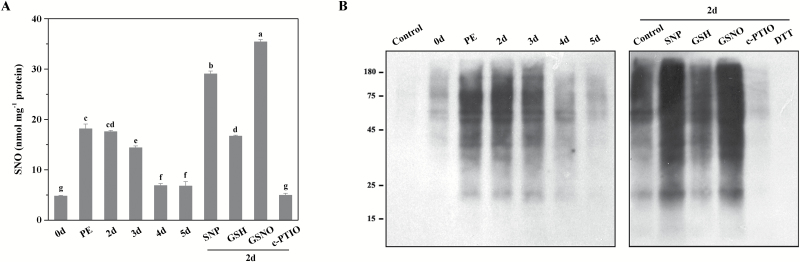
SNO and *S*-nitrosylated protein levels in the seeds during controlled deterioration. (A) SNO content of the seeds during controlled deterioration and the effect of pharmacological treatments on the SNO levels in the seeds after 2 d of CDT. Data are the mean ±SD of three replicates. *P<0.05; **P<0.01. (B) Change of *S*-nitrosylated protein levels during seed ageing and the effect of reagent treatment on protein *S*-nitrosylation in the seeds after 2 d of CDT. *S*-Nitrosylation was detected using a biotin-switch assay with anti-biotin antibody. PE, post-equilibration.

Since *S*-nitrosylation is a fundamental NO-derived post-translational modification to transduce NO bioactivity, we questioned if it would function in seed ageing. Thus, the contents of proteic-SNO groups and protein *S*-nitrosylation in aged seeds were analysed. GSNO, a natural *in vivo* reservoir of NO, was used to induce *S*-nitrosylation, and GSH served as a control. The results of the Savile–Griess assay illustrated that the SNO level was greatly induced and peaked at the beginning of ageing, reaching nearly 3.7-fold the content in non-aged seeds (Fig. 6A). It then decreased rapidly over time, but was still higher than that in the non-aged control. Pre-treatment with SNP or GSNO further increased the SNO contents of the 2 d aged seeds, reaching 1.7- and 2.0-fold of those in the 2 d aged seeds that were not treated, respectively. Since exposure to GSH did not change the SNO content in 2 d aged seeds, the effect of the GS group in GSNO was excluded. The addition of c-PTIO markedly reduced the SNO level to only 28.2% of the level in 2 d aged seeds, which was probably due to decreased endogenous NO. All these data illustrated that NO increased the contents of proteic-SNO groups during CDT.

When the total proteins from ageing seeds were subjected to a biotin-switch assay, the levels of the *S*-nitrosylated proteins were consistent with the SNO contents (Fig. 6B). During CDT, the *S*-nitrosylated proteins increased at the beginning of ageing and then decreased. SNP or GSNO treatment increased the *S*-nitrosylation levels in 2 d aged seeds, but c-PTIO significant reduced it. As a negative control of GSNO, the GSH treatment did not obviously change the level of *S*-nitrosylation. Protein *S*-nitrosylation could not be detected in the DTT-treated sample, illustrating the specificity of the biotin-switch method to detect *S*-nitrosylated proteins. Taken together, these data indicate that the increase of *S*-nitrosylation results from elevated NO, and it may participate in the regulation of seed deterioration.

### Identification of *S*-nitrosylated proteins

**Fig. 7.**
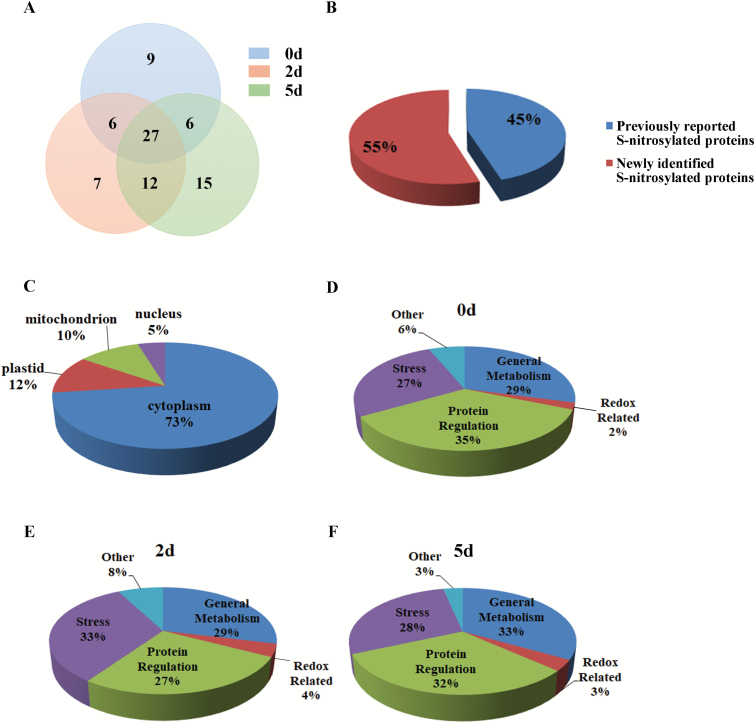
Analysis of *S*-nitrosylated proteins identified from ageing seeds using HPLC-MS/MS. (A) Overlap of the *S*-nitrosylated proteins in the seeds subjected to CDT for 0, 2, and 5 d displayed by a Venn diagram. (B) Proportion of *S*-nitrosylated proteins found in previous studies and newly identified in this study. (C) Predicted subcellular localization of the *S*-nitrosylated proteins identified from seeds subjected to CDT for 0, 2, and 5 d. (D–F) Functional categorization of the *S*-nitrosylated proteins identified from seeds subjected to CDT for 0 (D), 2 (E), or 5 d (F).

To identify *S*-nitrosylated proteins, biotinylated proteins were enriched and purified from non-aged seeds (0 d), and seeds in early (2 d) and later stages of ageing (5 d) using the biotin-switch method, and the proteins were screened using the HPLC-MS/MS proteomic approach. A total of 82 putative *S*-nitrosylated proteins have been identified in non-aged (48), 2 d aged (52), and 5 d aged (60) seeds (Fig. 7A; Supplementary Table S3). Among them, 33% (27/82) of proteins were constitutively *S*-nitrosylated in all three samples, and 41% (34/82) proteins were *S*-nitrosylated only after ageing. In addition, 45% (37/82) of the unique *S*-nitrosylated proteins identified in this study were previously reported to have been *S*-nitrosylated (Fig. 7B; Supplementary Table S3), indicating the reliability of our results. GO subcellular component analysis revealed that the *S*-nitrosylated proteins were significantly enriched in the cytoplasm (73%), followed by the plastid (12%), the mitochondrion (10%), and the nucleus (5%) (Fig. 7C). GO biological process analyses revealed that approximately ~35% of the *S*-nitrosylated proteins functioned in protein regulation, 29% in general metabolism, 27% in stress, and 2% in redox homeostasis in non-aged seeds (Fig. 7D). In aged seeds, the GO results changed, with more proteins related to the stress response and redox homeostasis *S*-nitrosylated (Fig. 7E, F).

### *S*-Nitrosylated proteins enriched in glycometabolism

It is noteworthy that the GO categorization of *S*-nitrosylated proteins revealed that 11 proteins are involved in carbohydrate metabolism. Of these proteins, phosphoglycerate kinase (PGK) in glycolysis and succinyl-CoA ligase [ADP-forming] subunit beta (SUCLG) in the tricarboxylic acid (TCA) cycle are *S*-nitrosylated during the early stage of ageing. The *S*-nitrosylation of these proteins could serve as a signal to initiate ageing. The glycolytic enzyme 2,3-bisphosphoglycerate-independent phosphoglycerate mutase (PGAM-i) and the second reducing enzyme in the PPP, 6PGDH, are *S*-nitrosylated during the later stages of ageing. The modification of these proteins could be closely related to the final loss of seed vigour. TKL, the rate-limiting enzyme in the non-oxidative part of the PPP, was *S*-nitrosylated in the non-aged seeds, but this modification disappeared after ageing. This could occur because the *S*-nitrosylation of TKL is necessary for its normal biological function. Although three proteins in glycolysis (FBA, TPI, and GAPDH) and one enzyme in the TCA cycle (MDH) were detected in both non-aged and ageing seeds, their *S*-nitrosylation levels may differ. These results highlight a possible role for these *S*-nitrosylated proteins in the regulation of seed deterioration. Since NO can cause metabolites involved in carbohydrate metabolism to change during seed ageing, but their gene expression was not affected by NO, we hypothesized that this post-translational modification, the *S*-nitrosylation of essential proteins in glycolysis, the TCA cycle, and the PPP potentially regulated carbohydrate metabolism during seed deterioration.

### Seed deterioration and *S*-nitrosylation regulate the activities of the enzymes in carbohydrate metabolism

**Fig. 8.**
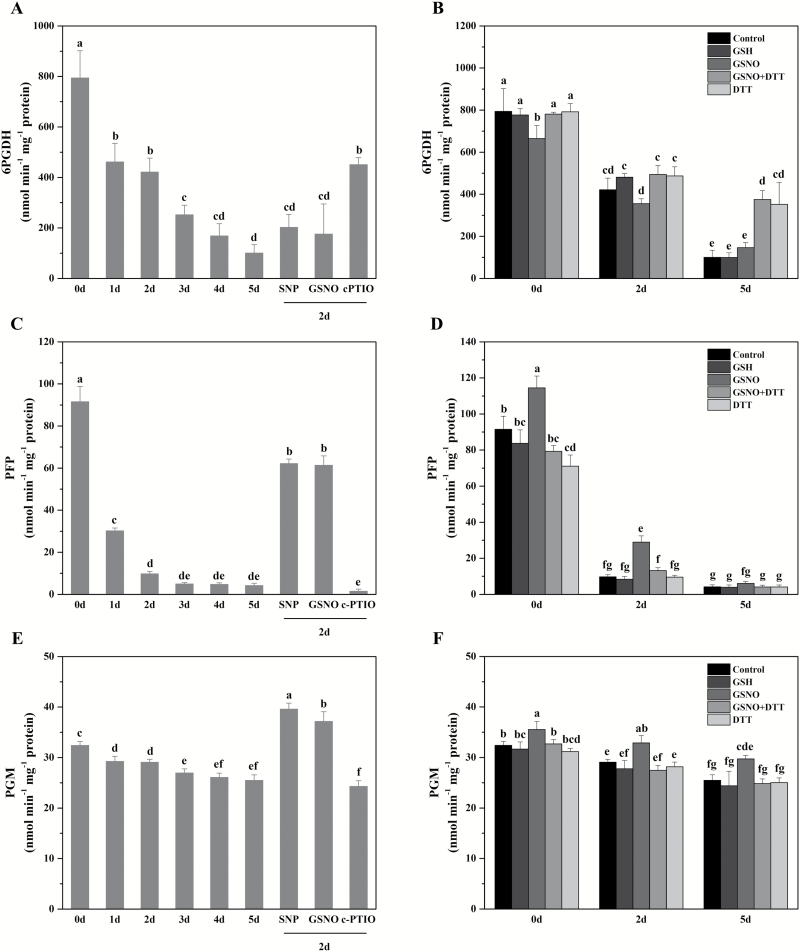
Effect of seed ageing and protein *S*-nitrosylation on enzymatic activity. (A, C, E) Effects of the CDT and NO on the enzymatic activity of 6PGDH (A), PFP (C), and PGM (E). Seeds are subjected to CDT for different times or pre-treated with 100 μM SNP, 100 μM GSNO, or 50 μM c-PTIO and then subjected to CDT for 2 d. The crude extracts were used for activity assays. Data are the mean ±SD of three replicates. Different superscript letters indicate significant differences at the *P*<0.05 level. 6PGDH, 6-phosphogluconate dehydrogenase; PFP, pyrophosphate-fructose 6-phosphate 1-phosphotransferase; PGM, phosphoglucomutase. (B, D, F) Effects of the GSH and GSNO treatments on the enzymatic activity of 6PGDH (B), PFP (D), and PGM (F) in ageing seeds. Crude extracts of aged elm seeds were treated with 250 μM GSH, 100 μM GSNO, 100 μM GSNO plus 10 mM DTT, or 10 mM DTT, and used for activity assays.

To gain insight into the exact roles of protein *S*-nitrosylation in carbohydrate metabolism during seed deterioration, the enzyme activities were assayed in ageing seeds and the seeds treated with NO compounds. 6PGDH was *S*-nitrosylated in the later stage of ageing, and its activity dramatically decreased during ageing (Fig. 8A). SNP or GSNO treatment before 2 d of ageing decreased its activity, while c-PTIO had no significant effect. When the crude extracts of aged elm seeds were treated *in vitro*, GSNO inhibited its activity in non-aged and 2 d aged seeds, and it could be restored by the addition of DTT (Fig. 8B). The absence of an effect of GSNO treatment following the exposure to GSH confirmed the specificity of this post-translational modification. In contrast, 5 d aged seeds treated with GSNO have no significant effect on enzyme activity, which could be because this protein was already *S*-nitrosylated *in vivo* during the later stage of ageing. However, DTT addition markedly increased the activity of 6PGDH, suggesting that the inactivation of 6PGDH by protein *S*-nitrosylation and oxidative damage was blocked by 10 mM DTT. Taken together, we hypothesized that the inactivation of 6PGDH activity at the later stage of ageing was dependent on its *S*-nitrosylation.

PFP, the rate-limiting enzyme in the regulation of the primary carbohydrate metabolic flux towards glycolysis or gluconeogenesis, was *S*-nitrosylated in non-aged and 5 d aged seeds, and its activity decreased remarkably during ageing (Fig. 8C). Pre-treatment of the seeds with SNP or GSNO doubled its activity, while c-PTIO eliminated it. The GSNO treatment of the extracts of non-aged and 2 d aged seeds significantly increased the activity of the enzyme (Fig. 8D), and this effect was reversed in the presence of DTT. The addition of DTT alone can inhibit PFP activity in non-aged seed extracts but has no effect on aged seeds. This could be attributed to the non-reversible thiol modification of PFP in 2 d aged seeds and the irreversible *S*-nitrosylation or degradation of this protein in 5 d aged seeds. Taken together, PFP activity was reduced as ageing proceeds, but NO-dependent *S*-nitrosylation can delay it at the early stage of ageing.

PGM, an enzyme important in the allocation of carbon between polysaccharide formation and energy production, was *S*-nitrosylated in non-aged and 5 d aged seeds, and its activity gradually decreased during ageing (Fig. 8E). SNP or GSNO increased its activity, and c-PTIO decreased it. Exposure to GSNO significantly raised its activity in the crude extracts of non-aged and aged seeds (Fig. 8F). The addition of DTT to the GSNO-treated extracts counteracted the activation effect of GSNO on enzyme activity, but DTT treatment alone had no significant effect. These results showed that the PGM activity in ageing seeds was activated by modification of *S*-nitrosylation.

FBA, TPI, and MDH were *S*-nitrosylated in both non-aged and aged seeds, and TKL was only *S*-nitrosylated in non-aged seeds. During seed deterioration, the activity of TPI, MDH, and TKL decreased during ageing (Supplementary Fig. S4C, E, G). The activity of FBA increased first and decreased later (Supplementary Fig. S4A), and the initial increase in FBA activity might be a response to stress. *In vivo* and *in vitro* assays illustrated that the activities of these four enzymes were not regulated by NO-dependent *S*-nitrosylation (Supplementary Fig. S4).

## Discussion

NO is involved in many physiological processes in plants and plays important roles in the adaptation of plants to diverse growth conditions (Yu *et al.*, 2014). However, the role of NO in seed deterioration remains to be determined. In this study, we found that SNP enhanced the NO level and slowed the rate of seed deterioration, which was consistent with studies in the leaves of NOD transgenic Arabidopsis (Mishina *et al.*, 2007) or the *noa1* mutant (Guo and Crawford, 2005), suggesting a protective role for NO in the senescence of plants. In some cases, NO inhibited cell growth and caused cell death and senescence (Leshem and Haramaty, 1996; Pedroso *et al.*, 2000; Saviani *et al.*, 2002). Considering that the effect could be different if the type of NO donors and its concentrations differ (Murgia *et al.*, 2004), GSNO, l-arginine, and nitrite were also used, and they all have protective roles in seed deterioration like SNP (data not shown).

The NO level in the cotyledon was low before CDT but quickly rose to a high level at an early stage of ageing and then decreased from the third day of ageing. This pattern was consistent with the NO levels during the pre-harvest deterioration in developing soybean seeds identified by Wang *et al.* (2012). The early burst of NO could be a stress response of the seeds to the ageing treatment, and the later decrease of the NO level was probably due to the reduction of nitrite-dependent NO production (Wang *et al.*, 2012).

We next dissected how this signal molecule functions during deterioration. At transcriptional levels, the rate-limiting gene of glutathione synthesis, *γGCS*, increased at the early stage of ageing and then decreased. This might be responsible for the increase of GSH at the early stage of ageing (Hu *et al.*, 2012; Fig. 4E). We also found that *γGCS*, *GS*, and *GST* were activated after NO treatment, and this was consistent with the results in animal cells (Gegg *et al.*, 2003; Wesseling *et al.*, 2007). In Arabidopsis, the genes encoding GST were up-regulated following SNP treatment (Parani *et al.*, 2004). In *Medicago truncatula* roots, the expression of the *GCL* and *GS* genes induced by NO was correlated with the accumulation of the end-product GSH (Innocenti *et al.*, 2007). These results indicated that NO was a common regulator of glutathione metabolism. Previous research showed that the ROS burst may induce downstream PCD events (Wang *et al.*, 2015), and the conversion of GSH to GSSG was involved in the scavenging of the ROS generated during seed deterioration (Hu *et al.*, 2012). Several studies indicated that NO possessed the ability to counteract the generation of ROS (Wang *et al.*, 2014). However, whether NO functions in seed ageing is not clear. Our data provided evidence that NO stimulates GSH synthesis in seed deterioration, which could reduce ROS production and subsequent cell death.

Previous studies reported that NO could modulate methionine metabolism and subsequent ethylene production. Zago *et al.* (2006) found that the expression level of *SAMS* increased with SNP treatment. In our study, we found that the contents of four metabolites and two genes (*MetE* and *SAMS*) in methionine metabolism decreased during CDT and increased after NO treatment, suggesting that NO might counteract the alleviation of methionine metabolism during seed deterioration. Since SAMS catalyses the synthesis of the ethylene precursor, and NO is known to regulate the biosynthesis of ethylene, this enzyme probably mediates the crosstalk between ethylene and NO signalling. Lin *et al.* (2013) found that the expression of *ACS2* was up-regulated by applying SNP to salinity-stressed seeds of Arabidopsis, and the germination inhibition caused by H_2_O_2_ was eliminated by the application of ACC (1-aminocyclopropane-1-carboxylic acid). In our study, we found that the down-regulation of *ACS* during seed deterioration was reversed by NO, which illustrated that NO might alleviate oxidative stress-induced cell death via an increase in the ethylene pathway.

In pea seeds, the genes encoding a putative GSNOR were up-regulated after ageing, implying a participation of *S*-nitrosylation in seed deterioration (Chen *et al.*, 2013). In our study, SNO and *S*-nitrosylated proteins accumulated at the early stage of ageing, suggesting that NO-induced *S*-nitrosylation could participate in the regulation of seed deterioration. After 3 d of ageing, the *S*-nitrosylated proteins exhibited a dramatic decrease, which was probably due to *S*-nitrosylation-facilitated protein degradation (Albertos *et al.*, 2015). A growing number of *S*-nitrosylated proteins have been identified in plants. Lindermayr *et al.* (2005) identified 63 *S*-nitrosylated proteins in GSNO-treated Arabidopsis cell cultures. In this study, we identified 82 candidate *S*-nitrosylated proteins in non-aged and ageing seeds. Eleven of these enzymes are involved in carbohydrate metabolism. Previous studies have shown that carbohydrate metabolic proteins are modified by *S*-nitrosylation during cold stress (Sehrawat *et al.*, 2013*b*) and in mouse liver (Doulias *et al.*, 2013). The *S*-nitrosylation of GAPDH promotes cell death in animals (Hara *et al.*, 2005; Foster *et al.*, 2009). However, GAPDH was *S*-nitrosylated in both non-ageing and ageing seeds, and its function in seed deterioration should be further studied.

In general, the activities of the enzymes in glycolysis, the TCA cycle, and the PPP were inhibited during ageing, and most of the associated metabolites were down-regulated. These results were consistent with other studies on seed deterioration showing that enzymes in glycolysis, PGAM-i, FBA, and GAPDH (Chen *et al.*, 2013), MDH in the TCA cycle (Wang *et al.*, 2012), and 6PGDH in the PPP (Chen *et al.*, 2013) were down-regulated after ageing. The decrease of glycolysis might lead to lower ATP production and NADH for oxidative phosphorylation. Our previous study also reported the loss of mitochondrial transmembrane potential and the decrease of mitochondrial ATP production after seed deterioration (Wang *et al.*, 2015). These results showed that aged seeds might not have sufficient energy supplies for their normal metabolism. It is known that the reduction of cellular energy levels can negatively affect senescence, and the inactivation of energy metabolic enzymes could lead to the disorder of mitochondrial function, lack of energy supply, and cell death (Jin *et al.*, 2014). Thus, we concluded that an insufficient energy supply may lead to cell death and loss of seed vigour during seed deterioration.

Both glycolytic enzymes PFP and PGM were activated by *S*-nitrosylation. These results were consistent with the increased levels of five metabolites upon SNP or GSNO treatment. Early studies found that NO can up-regulate the rate of glucose consumption, protecting the cells from ATP depletion and cell death. The glycolytic ATP also inhibited ATP synthase so as to maintain the mitochondrial membrane potential (Almeida *et al.*, 2001). In addition, the high glycolytic flux leads to selective diversion of carbon into several anabolic pathways and the maintenance of the signal transduction processes by changing the levels of metabolites (Warmoes and Locasale, 2014). Thus, *S*-nitrosylation may also alleviate cell death via glycolytic activation and consequent ATP supplementation.

We also found that the PPP was inactivated by *S*-nitrosylation. The PPP receives its substrate from glycolysis and feeds its products back into glycolysis, so the activity of this pathway is also important to determine the flux through glycolysis (Bettey and Finchsavage, 1996). Misiti *et al.* (2002) clearly explained that nitroglutathione affects the partitioning of glucose between glycolysis and the PPP. Thus, we concluded that protein *S*-nitrosylation inactivated PPP, and more glucose entered into the glycolysis flux. These results were also consistent with the *S*-nitrosylation-induced activation of glycolysis. In other studies, 6PGDH was insensitive to NO in Arabidopsis seedlings (de Freitas-Silva *et al.*, 2017), and MDH was inactivated by *S*-nitrosylation in pea leaves (Ortega-Galisteo *et al.*, 2012). This variation in results could probably be attributed to the different species and tissues used, as well as the type, concentrations, and the expiration time of the NO donors. Although G6PDH was generally considered to be the regulatory enzyme of the PPP, it was not *S*-nitrosylated during seed ageing. However, 6PGDH was modified and inactivated by *S*-nitrosylation during seed deterioration. It was also found to be important for the oxidative PPP and might function during multiple stress conditions (Hou *et al.*, 2007).

**Fig. 9.**
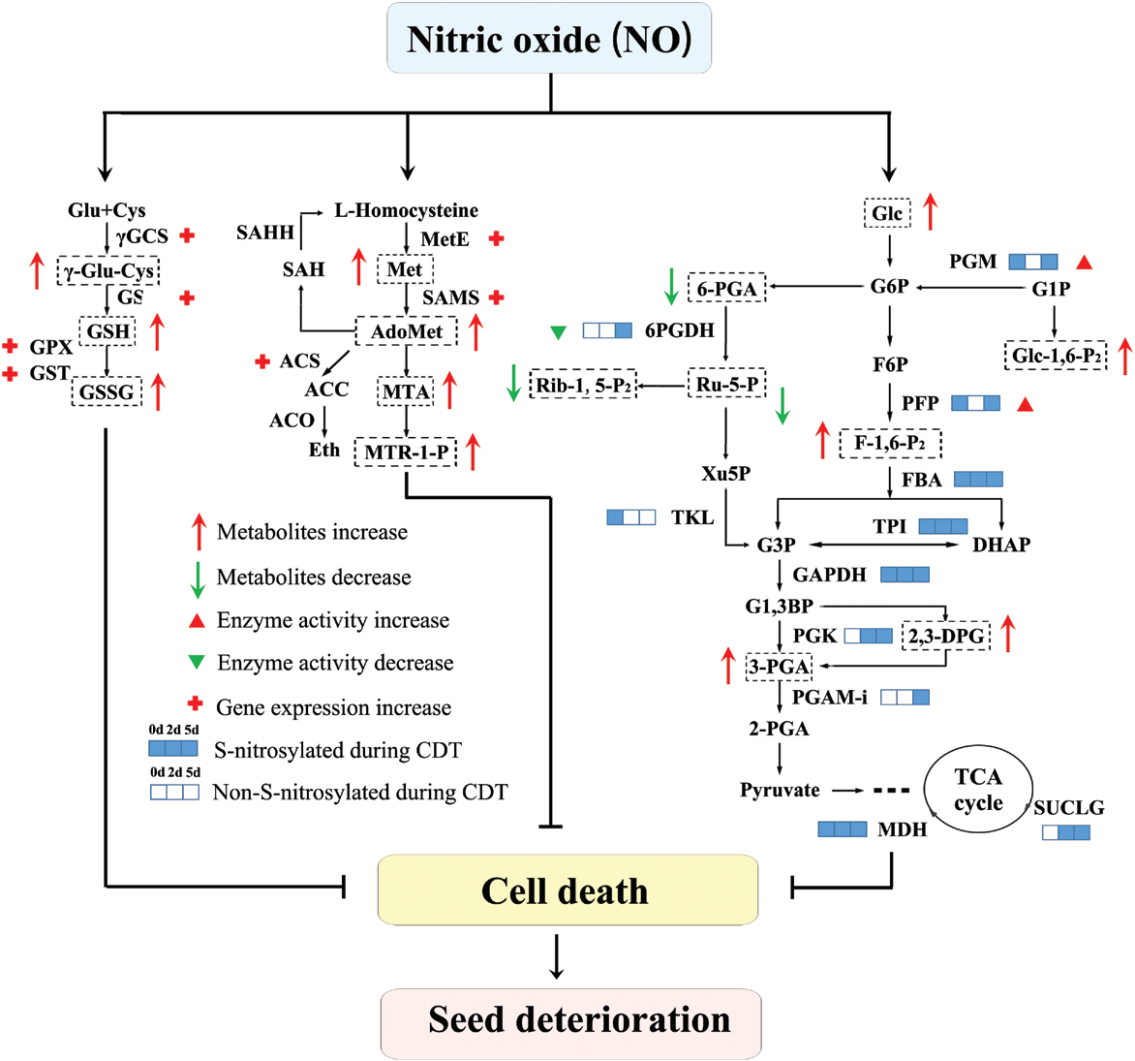
A diagram of the NO-induced metabolic changes during seed ageing. The process of CDT for 0, 2, and 5 d is illustrated using three linked squares. The square is filled blue if one protein was *S*-nitrosylated at the indicated time point of CDT, and the square remained blank if *S*-nitrosylation was not detected. An *S*-nitrosylation-mediated increase in enzyme activity is indicated with a red solid arrowhead, and the decrease in enzyme activity is indicated with a green filled arrowhead. Metabolites that are regulated by the NO treatment are bordered with dashed boxes. The increase in the metabolite levels following SNP or GSNO treatments is indicated with a red arrow, and the decrease of the metabolite levels is indicated with a green arrow. Genes up-regulated by NO are shown with red plus signs.

In summary, we found that NO can reduce the cell death and vigour loss during seed deterioration (Fig. 9). Glutathione and methionine were activated by NO at the transcriptional level and could reduce ROS production and cell death. NO-mediated protein *S*-nitrosylation in carbohydrate metabolism may induce the activation of glycolysis and the inactivation of the PPP, thus protecting the seeds from energy deficiency. A possible model is that NO activated glutathione, methionine, and glycolysis metabolism, protecting the seeds from the ROS burst and energy deficiency, thus alleviating cell death during seed deterioration.

## Supplementary data

Supplementary data are available at *JXB* online.

Fig. S1. Effect of the different concentrations of SNP on seeds subjected to CDT for 0, 3, and 5 d.

Fig. S2. OPLS-DA score plots derived from the UPLC-Q-TOF MS spectra following CDT.

Fig. S3. The expression pattern of genes in carbohydrate metabolism with different treatments using qRT-PCR.

Fig. S4. Change of enzyme activities in four *S*-nitrosylated proteins during seed ageing.

Table S1. Sequences of primers used in real-time RT-PCR.

Table S2. Concentrations of the metabolites regulated by NO in the seeds subjected to different treatments.

Table S3. *S*-Nitrosylated proteins in the seeds during controlled deterioration.

Supplementary Tables S1-S3

Supplementaty Figures S1-S4
