# Supplementary material for: Nitric oxide alleviates cell death through protein S-nitrosylation and transcriptional regulation during the ageing of elm seeds
Source: J Exp Bot. 2018 Jul 25;69(21):5141–55. doi: 10.1093/jxb/ery270 (PMC6184755; doi:10.1093/jxb/ery270)
Supplement: Supplementary Tables S1-S3 [file ery270_suppl_supplementary_tables-s1-s3.pdf]

Supplementary Table S1 Sequences of primers used in real-time RT-PCR.

Gene	Accession number	Sequences		Product size (bp)
		Forward	Reverse	
ACT	MH568818	TATAATGAATTGCGTGTAGCCC	CATAGCAGGAGTGTTGAAGGTC	126
γGCS	MH568835	ACATGACGGCAGATTGGACC	AGTCACCGCATTCAGGAACC	185
GS	MH568823	GATTCATACCCACAAGCCAAAAC	GTCACCAACGACAAGCCCAT	242
GST	MH568824	GGGCAGAAGTTGAGGCTCAT	GGAGGTGGTGAAGATCTGCC	216
GPX	MH568822	GTCGGTGGCGTCAAAGTTTT	CATTGGGAAGAAAGGACGGC	128
MetE	MH568827	TCCGGCTTGAATGTGCTTGT	ATCGTTGGCCCAGATGTTCC	207
SAMS	MH568832	GACGAGAAAACCATCTTCCACTT	AGCCCATTTGCTACGATACTCTT	221
SAHH	MH568831	GCCGGGAGTACAAGGTCAAG	TGGAGAGAGCCGGTGATTCT	154
ACS	MH568817	GGCACCACAAGAAGCTCCTT	TTCGCCTGGTTCTTCGTTCC	159
HXK	MH568825	GCTGCCGGGATTTTGGGAAT	ACTGGGAATGAGATGCAGCG	244
PGM	MH568829	TGAGAATGTGGATGCGGGTG	AAACCAGTCGCGATCCATCC	218
K6PF	MH568826	AGCAGTAGAAATGGCCCAGC	AAGCAGCAGTCCACATCTCG	153
FBA	MH568819	GACAAGGGCACAGTTGAGCT	TTGATGGCAAGCTGGGATGG	167
PFP	MH568828	ACCCATCGCTCACTTTCTCA	TGCTCTTTGGGTTGTGAAGC	170
TPI	MH568834	CTGATTTCCATGTTGCGGCC	GTCGATCCAGATTCCCGCTG	247
GAPDH	MH568821	GAATGGCTTTCCGTGTTCCTAC	CAGTTTCCCCTCAGACTCTTCCT	122
PYK	MH568830	ATCGTCTGCACTTTGGGACC	ACGGCGCAAAGAATACCAGT	173
G6PDH	MH568820	TGCAACCTTCAGAAGCCATGT	GCCGCCTTTAACTCGTCTCT	202
6PGDH	MH568816	GGAGATCATTGAGCGGCAGT	AATCACGTTGAGCCTGGACC	155
TKL	MH568833	TTGGGTGGCATGTGATCTGG	CCTCGGGTACATGGAAAGGC	246

ACT, actin; *G6PDH*, glucose 6-phosphate dehydrogenase.

Supplementary Table S2 Concentrations of the metabolites regulated by NO in the seeds subjected to different treatments.

CDT-，NO+

Adduct	RT(s)	m/z	Formula	Metabolite	p-value	2d/0d		5d/0d		SNP/2d		GSNO/2d		cPTIO/2d	
						Fold	VIP	Fold	VIP	Fold	VIP	Fold	VIP	Fold	VIP
M+Na-2H	2.57	912.62	C48H93NO11S	3-O-Sulfogalactosylceramide (d18:1/24:0)	1.06E-03	1.09		0.01	2.64	0.87	2.11	0.59	2.78	0.34	2.86
M-H, M+Na-2H	6.26	565.05	C15H24N2O17P2	Uridine diphosphategalactose	1.19E-10	0.82	3.21	0.06	6.13	0.93	2.46	0.58	4.93	0.48	4.48
M+Na-2H	3.97	280.99	C6H13O7PS	5-Methylthioribose 1-phosphate	1.41E-05	0.99		0.05		1.18		0.90		0.38	1.33
2M+FA-H	1.23	643.17	C10H13N5O6	8-Hydroxyguanosine	6.92E-04	0.56		0.03		0.84	1.27	0.71		0.14	
M+Na-2H	2.52	988.58	C48H89NO18	Trihexosylceramide (d18:1/12:0)	2.74E-03	1.04		0.02	1.80	0.76	2.13	0.70	1.57	0.49	1.70
M-H, M+Na-2H	1.23	933.20	C30H49N8O18P3S	Pseudoecgonyl-CoA	6.72E-05	0.56	1.25	0.01	1.66	0.92		0.85		0.39	1.13
M-H, M+Na-2H	6.07	606.07	C17H27N3O17P2	UDP-N-acetyl-D-mannosamine	7.63E-14	0.56	2.77	0.03	2.96	0.84	1.75	0.69	1.66	0.44	1.84
M+Na-2H	2.55	870.63	C50H92NO7P	PC(o-22:1(13Z)/20:4(8Z,11Z,14Z,17Z))	2.12E-03	1.02		0.02	2.43	0.77	2.50	0.51	2.72	0.40	2.42
M+FA-H	3.87	429.19	C19H25BN4O4	Bortezomib	4.72E-09	1.12	1.59	0.15		0.90	1.15	0.57	1.30	0.49	
M-H, M+Na-2H	1.76	479.12	C19H24N6O5S2	Cefepime	7.26E-03	0.60		0.00	1.11	0.70		0.65		0.26	1.05
M-H, M+Na-2H	2.35	257.03	C11H12Cl2N2O	Lofexidine	3.67E-03	1.25		0.37	1.20	1.39	2.06	0.76	1.27	0.48	2.38
M+ACN+H	4.17	947.27	C41H45O23+	Cyanidin 3-O-(2''-xylosyl-6''-(6'''-caffeoyl-glucosyl)-galactoside)	4.05E-08	1.28		0.05		1.30		0.84		0.37	1.16
2M+Na	3.67	347.05	C9H6O3	Umbelliferone	2.01E-09	0.70		0.17	1.01	0.84		0.58		0.41	
2M+H	13.46	785.03	C10H21AsO9S	beta-D-3-[5-Deoxy-5-(dimethylarsinyl)ribofuranosyloxy]-2-hydroxy-1-propanesulfonic acid	2.22E-16	1.93	1.71	0.04		0.66	1.39	0.59	1.27	0.11	2.11
2M+FA-H	3.06	133.01	BHO2	Boric acid (HBO2)	8.93E-04	0.79	1.96	0.11	2.48	1.14	1.97	0.80	3.24	0.40	5.08
M+ACN+H	4.20	801.23	C32H39O21+	Delphinidin 3-lathyroside 5-glucoside	2.03E-07	0.55	1.10	0.06	1.09	0.90		0.77		0.41	
2M-H	2.55	869.18	C20H19O11+	Delphinidin 3-arabinoside	5.59E-04	1.10		0.25	1.14	0.94		0.59	1.42	0.47	1.46
M+FA-H	0.51	481.10	C20H20O11	Homomangiferin	5.62E-10	1.04		0.51		0.75		0.63		0.45	1.03
M-H	1.45	465.10	C21H22O12	(-)-Epicatechin 3'-O-glucuronide	3.02E-03	1.44	1.45	0.49		0.73	1.19	0.67	1.21	0.48	1.70

2M+NH4	3.81	580.20	C12H15N3O3S	albendazole S-oxide	1.14E-08	1.07		0.05	1.07	1.46	1.05	0.80		0.45	
M+ACN+ H	4.83	166.06	C5H4N2O2	Pyrazin-2-carboxylic acid	1.32E-10	0.54		0.07	1.05	0.91		0.69		0.43	
M+ACN+ H	4.90	195.04	C3H7NO4S	3-Sulfinato-L-alaninate	1.23E-07	0.79	1.02	0.33	1.34	0.79	1.02	0.58	1.29	0.38	1.43
M+Na-2H, 2M+FA-H	3.45	773.25	C16H20N4O4S	hydroxytorsemide	1.80E-04	0.67	1.29	0.21	1.92	0.70	2.17	0.55	1.80	0.46	1.60
M+H, M+Na, M+K	4.31	280.09	C8H20NO6P	Glycerophosphocholine	2.70E-09	0.28	2.91	0.11	2.14	0.60		0.57		0.38	1.02
2M+FA-H	5.26	635.11	C8H14N3O7P	5-Aminoimidazole ribonucleotide	7.13E-11	0.41	1.86	0.02	1.72	0.93		0.66		0.26	1.06
2M+FA-H	1.39	395.19	C6H13N3O3	Argininic acid	9.73E-11	0.45	1.22	0.08		0.86		0.78		0.39	
M-H	5.26	611.14	C20H32N6O12S2	Oxidized glutathione	2.60E-11	0.42	4.32	0.03	4.12	0.95	1.55	0.69	1.56	0.30	2.49
M-H	1.30	837.15	C24H41N8O17P3 S	Beta-Alanyl-CoA	1.59E-03	0.07	1.90	0.02	1.37	0.59		0.77		0.35	
2M+FA-H	5.22	549.12	C8H16N2O3S2	Cysteinyl-Methionine	7.29E-04	0.44	1.05	0.07		0.74		0.63		0.32	
M+Na-2H	4.24	529.19	C22H36O13	6-O-Oleuropeoylsucrose	5.59E-06	0.46	1.12	0.08		0.72	1.09	0.64		0.46	
M-2H, M+Na-2H	5.26	655.10	C28H26O17	3'-(2",3"-Digalloylglucosyl)-phloroacetophenone	1.48E-11	0.46	1.91	0.03	1.86	1.08		0.77		0.33	1.15
2M+FA-H	4.21	593.12	C18H10O3	4-Phenyl-1H,3H-naphtho[1,8-cd]pyran-1,3-dione	6.23E-04	0.23	1.58	0.09	1.26	0.68		0.56		0.36	
M-H	5.27	583.02	C20H20N6O7S4	Cefodizime	1.73E-05	0.27	1.02	0.12		0.53		0.51		0.33	
M-H	1.78	330.12	C10H17N7O6	N'-Hydroxyneosaxitoxin	2.84E-03	0.28	2.18	0.16	0.00	1.29	0.00	0.51	1.10	0.36	
M+Na-2H	4.24	453.10	C18H24O12	Licoagroside B	1.31E-04	0.27	2.02	0.24	1.43	1.33	1.11	0.58		0.50	
M+NH4	3.12	572.20	C25H30O14	Lippioside II	7.74E-09	0.93		0.41		1.22		2.37	1.37	1.60	
M+NH4	1.56	391.19	C16H32O2	Isopalmitic acid	5.61E-05	0.48	2.60	0.88	2.10	1.89	1.18	2.42	1.13	1.82	1.11
M+H, 2M+H	9.32	270.28	C17H35NO	Capsiamide	1.87E-09	0.40	1.50	0.39		1.58		2.12	1.05	1.73	
M+Na-2H	2.34	271.04	C13H14O3S	2-[(5-Methylsulfinyl)-4-penten-2-ynylidene]-1,6-dioxaspiro[4.4]non-3-ene	1.83E-03	1.00		0.21		2.19	1.01	0.58		0.85	
M+H-2H2O	4.46	453.16	C25H28O10	Egonol glucoside	1.07E-14	1.42	1.27	0.67	1.08	2.09	2.63	1.28	1.28	1.19	

M+FA-H	1.66	485.15	C17H28O13	Propylene glycol alginate	2.69E-03	1.64	2.01	0.64		2.01	1.01	1.39	1.28	0.99	
2M+FA-H	1.05	689.30	C20H22N2S	Mequitazine	1.88E-03	0.39	2.86	0.25	0.00	5.30	1.39	1.65	0.00	1.57	
M+K	4.05	550.15	C20H33NO14	O-6-deoxy-a-L-galactopyranosyl-(1->2)-O-b-D-galactopyranosyl-(1->4)-2-(acetylamino)-1,5-anhydro-2-deoxy-D-arabino-Hex-1-enitol	7.07E-09	0.39	1.08	0.08		2.34	1.11	2.28		1.86	
M+ACN+H	4.35	844.25	C34H42O22	Brassicoside	8.01E-04	0.26	1.46	0.05	1.15	2.08		2.02		1.47	
2M-H	3.46	497.10	C8H13N2O5S-	Glutamyl-Cysteine	2.11E-03	1.07	1.03	0.62	1.03	1.30	1.25	1.19	1.15	0.61	1.52
2M+FA-H	3.47	659.16	C10H17N3O6S	Glutathione	4.01E-03	1.53	1.24	0.67	1.09	1.28	1.13	1.44	1.22	0.46	1.32
M-H	5.26	611.14	C20H32N6O12S2	Oxidized glutathione	2.60E-11	0.48	4.32	0.04	4.12	1.53	1.24	1.39	1.56	0.30	2.49
2M+FA-H	3.45	343.10	C5H11NO2S	L-Methionine	4.00E-06	1.01	1.24	0.64	1.48	2.42	1.77	2.19	1.05	0.87	1.63
M+Na-2H	3.87	420.12	C15H23N6O5S+	S-Adenosylmethionine	5.56E-05	0.76	1.30	0.05	1.27	1.56	1.77	1.39	2.11	0.70	2.14
2M-H	1.05	593.17	C11H15N5O3S	5'-Methylthioadenosine	2.82E-03	0.73	2.40	0.70	1.21	1.18	1.52	1.13	1.46	0.90	1.42
M+Na-2H	3.97	280.99	C6H13O7PS	5-Methylthioribose 1-phosphate	1.41E-05	0.70	1.62	0.21	4.30	1.88	4.04	1.50	2.38	0.82	1.33
M+Na-2H	9.97	360.97	C6H14O12P2	Alpha-D-Glucose 1,6-bisphosphate	4.84E-03	0.45	2.61	0.45	1.24	1.63	3.09	2.17	3.26	0.80	3.34
M+H-H2O	4.11	163.06	C6H12O6	D-Glucose	1.44E-07	0.93	1.18	0.44	1.11	1.47	1.27	1.40	1.12	0.06	1.05
M+Na-2H	1.29	286.93	C3H8O10P2	2,3-Diphosphoglyceric acid	1.15E-03	1.01	1.05	0.43	1.01	1.58	1.46	1.69	1.12	1.04	1.26
M+ACN+H	4.19	228.03	C3H7O7P	3-Phosphoglyceric acid	1.51E-03	1.19	2.15	0.38	1.94	2.85	1.07	2.72	1.43	1.04	1.47
2M-H	1.26	678.98	C6H14O12P2	Fructose 1,6-bisphosphate	9.89E-06	0.97	1.63	0.17	1.25	1.27	3.05	1.19	3.15	0.68	2.09

CDT-，NO-

Adduct	RT(s)	m/z	Formula	Metabolite	p-value	2d/0d		5d/0d		SNP/2d		GSNO/2d		cPTIO/2d	
						Fold	VIP	Fold	VIP	Fold	VIP	Fold	VIP	Fold	VIP
M+K	4.11	204.99	C8H6O4	Terephthalic acid	4.06E-04	0.49		0.12	1.24	0.38		0.56		1.56	
2M-H	2.56	578.91	C10H10O2S4	Bis(2-methyl-3-furanyl)tetrasulfide	3.34E-03	0.93		0.28		0.31	1.07	0.02		0.53	
M+ACN+H	1.78	818.59	C14H14INO2	3-Iodothyronamine	2.02E-13	0.28	3.08	0.01	1.94	0.13		0.45		0.87	
2M+FA-H	3.40	747.07	C14H13N3O4S2	Meloxicam	9.76E-03	0.76		0.34		1.16	1.34	1.46		2.12	1.29
M+ACN+H	4.05	356.08	C16H10O7	Laccaic acid D	3.03E-08	1.10		0.47	1.23	0.67	2.17	1.04		2.09	2.62

M+NH4, M+Na	1.17	436.18	C18H26O11	Oleoside dimethyl ester	2.55E-15	1.21	1.13	0.54		0.88		1.00		4.37	3.98
M-H	4.47	231.06	C15H8N2O	Sampangine	4.31E-03	1.55		0.48		1.03		1.40		2.10	1.10
M+Na-2H, M+FA-H	1.60	453.12	C22H24O9	Pinostrobin 5-glucoside	6.33E-06	1.52	1.02	0.64		1.05		1.09		3.94	2.45
M+H-2H2O	5.56	238.96	C6H11O6PS2	Malathion dicarboxylic acid	1.18E-07	1.95		0.09		0.63		1.79		3.50	1.19
M+FA-H	2.68	359.04	C16H11ClN2O3	Clorazepate	4.45E-03	0.25	2.17	0.03	2.02	1.76	1.72	1.84	1.41	2.32	1.68
2M+FA-H	1.12	925.41	C22H32O9	3'-Hydroxy-HT2 toxin	1.61E-03	0.11		0.02	1.08	0.76	1.94	0.64	1.47	2.02	1.06
M+Na-2H	1.19	417.14	C16H28O11	1-(3-Methylbutanoyl)-6-apiosylglucose	1.73E-04	0.35		0.18		1.01		1.06		5.95	1.04
2M+K	5.59	673.24	C16H19N3O4	Tryptophyl-Hydroxyproline	1.65E-05	0.83		0.06	1.05	0.61		0.23	1.16	0.55	
M-H	3.35	665.21	C24H42O21	Fagopyritol B3	4.85E-03	0.56	2.21	0.17	2.51	0.78	1.94	0.44	2.37	0.69	1.62
2M-H	0.44	715.17	C15H18O10	Dihydrocaffeic acid 3-O-glucuronide	4.60E-05	1.44	1.39	0.19	1.35	1.17		0.49	1.22	0.52	1.39
2M-H	1.12	667.28	C16H19FN4O3	Amifloxacin	3.86E-05	0.55	1.31	0.13	1.14	0.73		0.47		0.61	
2M+FA-H	5.60	733.20	C17H16N2O6	dehydronifedipine	7.67E-07	0.54	1.35	0.19	1.54	0.83	1.21	0.47	1.59	0.66	1.34
2M-H	5.07	715.07	C17H12Cl2N4O	1'-hydroxytriazolam	2.53E-08	0.54	1.08	0.19	1.02	0.57		0.47		0.54	
2M-H	1.30	593.16	C13H15NO7	Hexahydro-6,7-dihydroxy-5-(hydroxymethyl)-3-(2-hydroxyphenyl)-2H-pyrano[2,3-d]oxazol-2-one	4.50E-03	0.01	1.05	0.01		0.56		0.49		0.58	
M+FA-H	2.22	282.08	C8H15NO7	N-(1-Deoxy-1-fructosyl)glycine	4.93E-03	0.25	1.21	0.37		0.52		0.13		0.84	
2M+K	2.57	320.99	C4H9Cl2N	Nornitrogen mustard	4.21E-03	0.36	1.60	0.22	1.32	0.63		0.12		0.94	
M+Na	4.30	365.10	C12H22O11	Sucrose	5.28E-07	0.99	1.83	0.70	2.00	0.78	2.47	0.85	2.16	1.00	1.91
M-H	3.62	275.02	C6H13O10P	6-Phosphogluconic acid	2.88E-03	0.91	1.13	0.04	3.56	0.21	5.20	0.17	1.45	0.93	2.46
M+NH4	1.19	328.02	C5H12O11P2	Ribose 1,5-bisphosphate	4.42E-03	0.69	1.40	0.43	1.20	0.73	1.21	0.68	1.20	0.88	1.08
2M+FA-H	1.39	505.04	C5H11O8P	D-Ribulose 5-phosphate	2.90E-03	0.72	1.71	0.60	1.24	0.47	1.10	0.65	1.44	1.18	1.05

CDT+，NO+

Adduct	RT(s)	m/z	Formula	Metabolite	p-value	2d/0d		5d/0d		SNP/2d		GSNO/2d		cPTIO/2d	
						Fold	VIP	Fold	VIP	Fold	VIP	Fold	VIP	Fold	VIP
M+Na-2H	1.61	267.07	C8H14N4O5	N2-Oxalylarginine	1.05E-08	3.71	2.55	3.75	1.47	0.98		0.55	1.18	0.49	1.41
M-H	0.85	435.13	C21H24O10	Phlorizin	3.17E-09	2.10	1.21	2.09		0.93		0.66		0.42	

M+Na-2H	2.12	872.15	C26H44N7O17P3S	Pentanoyl-CoA	4.14E-03	1.32		19.87	1.22	0.81		0.89		0.48	
M+NH4	3.47	349.13	C16H17N3O3S	5'-O-Desmethyl omeprazole	6.17E-06	3.77		8.02		1.88		2.71	1.11	1.73	
M+NH4	2.93	437.16	C25H22ClNO3	Fenvalerate	1.47E-11	2.33	1.23	3.41	1.43	1.86	1.57	5.71	3.99	1.98	1.41
M+Na-2H	0.75	179.03	C7H10O4	xi-2,3-Dihydro-3,5-dihydroxy-6-methyl-4H-pyran-4-one	2.80E-03	0.72		2.77	1.01	1.12		2.26	1.12	1.54	
2M-H	1.36	696.85	C9H11Cl3NO3PS	Chlorpyrifos	3.62E-03	0.59		2.72	1.07	1.18		2.49		1.86	
M+Na	1.24	535.23	C22H40O13	Ethyl 3-hydroxyoctanoate O-[glucosyl-(1->6)-glucoside]	1.73E-10	2.18	1.33	1.89		2.39	2.39	1.85	1.55	1.62	1.21
M+Na-2H	0.98	153.02	C5H8O4	(S)-2-Acetolactate	1.78E-09	2.03		3.57		2.77	1.12	2.80		1.89	
M+Na	1.78	763.39	C10H20O7	2,3-Butanediol glucoside	2.61E-11	2.37	1.29	4.66	1.35	2.32	1.46	2.89	1.16	1.15	

CDT+，NO-

Adduct	RT(s)	m/z	Formula	Metabolite	p-value	2d/0d		5d/0d		SNP/2d		GSNO/2d		cPTIO/2d	
						Fold	VIP	Fold	VIP	Fold	VIP	Fold	VIP	Fold	VIP
2M-H	0.99	423.11	C12H8N2O2	Questiomycin A	2.38E-05	6.58		14.89	1.00	0.50	1.08	0.56		1.70	
2M-H	0.72	615.46	C19H32O3	Annosquamosin B	7.41E-11	7.13		34.80	1.08	0.39		0.58		1.78	
2M+FA-H	1.00	601.52	C19H34O	2-Pentadecylfuran	1.40E-03	6.75		19.02	1.02	0.34		0.96		1.95	
M-H	9.69	585.49	C38H66O4	Erythrinasinate A	6.22E-15	9.67	2.84	21.11	2.57	0.41	3.08	0.68	2.07	1.54	2.06
M-H, M+Na-2H	0.71	565.48	C40H64	15-cis-Pytoene	9.60E-05	22.06	1.22	51.13	1.08	0.42		0.65		1.82	
M+H	2.97	504.34	C11H15N5O3S	5'-Methylthioadenosine	6.39E-10	2.03	2.40	1.82	1.21	0.44	1.52	0.70	1.46	0.62	1.42
2M-H	1.02	809.45	C21H31N3O5	Lisinopril	2.64E-03	3.48		4.35		0.35	1.35	0.52		0.57	
M-H, M+Na-2H, M+FA-H	0.70	885.69	C54H96O6	TG(15:0/18:3(9Z,12Z,15Z)/18:1(9Z))	4.70E-03	4.92	1.54	8.95	1.28	0.44		0.58		1.38	
M+Na-2H, 2M-H	0.70	567.18	C17H16O4	Stercurensin	3.35E-04	5.55		26.49	1.00	0.47		0.52		2.15	
2M+FA-H	0.70	363.21	C8H17NS	(Â±)-2-Pentylthiazolidine	1.06E-08	24.81		181.80	1.66	0.47		0.64		3.14	1.29
2M+FA-H	0.77	337.23	C7H16NO2+	1-Nitroheptane	1.31E-07	9.08	1.35	18.96	1.18	0.12		0.09		1.46	
2M+FA-H	0.98	563.26	C11H19N2O5-	Glutamyl-Leucine	3.68E-04	3.52		7.29		0.18	1.14	0.16		0.64	

M-H, M+Na-2H	0.70	609.51	C37H70O6	Glycerol 1,3-didodecanoate 2-decanoate	1.29E-08	9.79	1.18	29.57	1.10	0.29		0.19		1.71	
M+Na-2H	0.80	309.20	C16H32O4	(S)-10,16-Dihydroxyhexadecanoic acid	9.19E-07	27.15		233.37	1.35	0.11		0.18		1.94	
2M-H	0.90	571.42	C16H30O4	16-Hydroxy-10-oxohexadecanoic acid	6.11E-06	3.32		10.22	1.10	0.28	1.09	0.16		1.37	
M+FA-H	0.99	302.18	C17H23NO	Dextrorphan	1.54E-08	11.27	1.62	29.28	1.86	0.28	1.93	0.14	1.40	1.93	1.21
2M+FA-H	0.74	559.40	C14H27NO3	N-Lauroylglycine	9.01E-04	9.99	1.09	15.70		0.42		0.03		1.14	
2M+FA-H	0.74	365.27	C8H18NO2+	Propionylcholine	3.52E-06	8.68	3.77	14.57	2.90	0.38	2.15	0.16	2.21	1.42	1.69
2M-H	10.33	735.46	C19H32N2O5	Perindopril	1.70E-08	6.54	1.44	6.10		0.39	1.60	0.44	1.40	1.41	
M-H, 2M+FA-H	0.73	173.14	C9H20NO2+	(2S,4R,5S)-Muscarine	1.24E-03	18.57	2.17	30.82	1.63	0.30	1.27	0.11	1.26	1.70	1.25
M-H	10.29	692.45	C42H63NO7	Spirolide B	8.83E-10	1977.38	1.38	1731.81		0.15	1.70	0.09	1.62	1.39	
M+Na-2H, M+FA-H	1.00	573.35	C33H52O5	Tsugaric acid B	4.41E-03	4.79	1.16	7.32		0.39	1.25	0.29	1.06	1.78	1.09
M+Na-2H	1.28	503.19	C24H34O10	3'-Hydroxy-T2 Toxin	6.49E-06	9.89	2.55	7.22	1.22	0.11	1.71	0.15	1.47	1.17	
2M+FA-H	1.20	533.22	C15H16O3	Glandulone B	5.03E-06	5.44	1.47	3.35		0.37		0.32		0.88	
2M+FA-H	1.26	505.19	C14H14O3	Demethylbatatasin IV	1.32E-04	11.48	1.71	6.82		0.12	1.13	0.06	1.04	1.00	
M-H	0.90	243.03	C10H12O5S	4-phenylbutanic acid-O-sulphate	1.74E-04	2.78		4.33		0.33	1.01	0.28		0.74	
M+H, M+Na	1.62	417.13	C19H29IO2	Iophendylate	4.08E-03	0.77		2.72	1.50	0.48	1.44	0.40	1.29	0.92	
2M-H	1.00	864.73	C9H9I2NO3	3,5-Diiodo-L-tyrosine	1.25E-05	26.37		153.98	1.12	0.16		0.48		2.17	
M+FA-H	0.71	577.42	C37H56O2	2-Hexaprenyl-6-methoxyphenol	2.48E-04	15.07	1.01	36.99		0.34		0.28		2.03	
2M+H	9.33	585.36	C16H24N2O3	Carteolol	1.11E-16	8.98	1.00	31.13	1.06	0.42		0.36		2.93	1.30
M+FA-H	1.03	769.42	C48H56N2O4	Bismurrayafoline E	3.20E-06	10.80	1.85	28.23	1.40	0.26	1.11	0.10	1.07	2.31	1.34
M+Na-2H	0.70	883.68	C56H94O6	TG(14:0/20:5(5Z,8Z,11Z,14Z,17Z)/20:2n6)	5.55E-09	157.69	1.11	433.58	1.06	0.25		0.30		2.14	
M+Na-2H, M+FA-H	0.75	379.19	C20H30O4	12-Keto-leukotriene B4	2.48E-06	1.60		6.09	1.02	0.33		0.28		2.08	
M+H	3.51	182.08	C9H11NO3	L-Tyrosine	3.55E-11	2.04		5.08	1.31	1.42		1.22		3.07	1.66

2M-H	0.71	347.22	C6H14N4O2	L-Arginine	1.60E-05	2.21	1.55	5.17	1.80	0.72		0.93		2.20	1.68
M+H-2H2O	1.27	194.02	C5H12NO7P	5-Phosphoribosylamine	1.65E-04	23.74		75.73	1.37	0.67		1.56		2.82	1.04
M+Na-2H	0.90	355.10	C15H18N4O5	Mitomycin	2.93E-14	4.88	2.35	14.71	2.50	1.08		1.44	1.00	2.08	1.70
M+H	3.71	361.20	C17H24N6O3	Tryptophyl-Arginine	1.50E-06	49.52		556.39	1.25	0.88		0.82		5.80	
2M+FA-H	0.95	701.52	C19H36O4	Avocadene 2-acetate	2.60E-09	2.26		11.78	1.11	0.82		0.61		2.30	
M+Na-2H	0.72	293.21	C16H32O3	(R)-2-Hydroxyhexadecanoic acid	1.64E-08	26.12	2.49	213.43	4.11	0.76		0.88		3.07	2.43
M+H, M+K	0.92	631.31	C37H52O6	Avenestergenin B2	1.84E-11	2.76	1.18	7.69	1.58	0.67		0.69		2.48	1.53
M+FA-H	0.70	663.39	C39H54O6	3-O-p-trans-Coumaroylalphitolic acid	5.69E-13	2.24		12.73	1.75	0.70		1.17		2.42	1.06
2M-H	1.93	367.24	C14H16	1,4-Dimethyl-7-ethylazulene	3.15E-03	2.25		4.54		1.60	1.37	1.97	1.29	2.08	1.45
2M+Na	0.94	755.40	C20H30O6	Sporotrichiol	1.63E-07	23.35		154.37	1.17	1.39		1.54		4.64	1.07
M+H-2H2O, M+H	0.89	505.39	C31H52O5	Ganoderiol G	3.24E-14	2.11	4.33	5.98	4.99	0.90	3.39	0.82	2.87	2.26	4.75
2M-H	1.29	151.02	C2H4O3	Glycolic acid	1.41E-10	6.26	2.50	9.75	2.22	1.23	1.66	0.74	1.22	2.23	2.26
M+Na-2H	1.39	180.06	C7H13NO3	Valerylglycine	6.12E-08	2.07	3.30	3.50	3.54	1.33	3.32	1.77	3.73	2.56	4.39
M+H	1.03	674.48	C36H68NO8P	PC(14:1(9Z)/14:1(9Z))	3.39E-12	3.30	5.57	5.24	4.31	0.52	5.86	0.60	3.99	2.03	5.30
M+Na	1.44	257.99	C3H11NO7P2	Pamidronate	7.20E-13	2.11	2.75	3.90	3.08	1.26	2.11	1.59	2.74	2.01	3.27
M+FA-H	0.87	197.03	C8H8OS	S-Methyl benzenecarbothioate	4.64E-05	2.50		2.76		0.97		0.98		7.37	2.28
2M+K	0.91	633.32	C16H27NO2S	2-(4-Methyl-5-thiazolyl)ethyl decanoate	4.32E-08	2.51	1.03	4.82	1.14	0.52	1.02	0.51		2.02	1.16
M-H	3.35	182.07	C11H9N3	2-Amino-a-carboline	4.49E-03	10.98		16.13		0.87		1.07		2.11	1.41
M-H	1.14	139.02	C7H8OS	2-Propanoylthiophene	1.85E-04	2.31		2.67		1.35		1.36		8.72	2.15
M+Na-2H	1.68	603.40	C37H58O5	Hericene B	5.29E-03	2.82	1.06	1.96		0.63		0.76		2.04	
M+Na-2H	4.17	189.00	C5H4N4O3	Uric acid	1.03E-06	1.40		3.95	1.11	1.52	1.06	1.86		2.13	
M+Na-2H	0.94	196.02	C6H9NO5	N-Acetyl-L-aspartic acid	4.16E-03	1.47		3.22	1.17	1.40	1.23	1.72	1.12	2.09	1.13
M-H	4.55	274.14	C11H21N3O5	L-a-glutamyl-L-Lysine	9.32E-11	1.27		7.77	1.14	0.87		0.76		4.42	
2M-H	1.74	821.73	C25H49NO3	Tricosanoylglycine	1.05E-06	0.91		7.61	1.78	0.81		0.69		2.40	
M+NH4	0.96	227.16	C9H15N5O	Minoxidil	8.45E-12	1.47		3.08		0.95		0.98		2.71	1.07

M+H-2H2O	3.45	268.04	C10H11ClFN5O3	Clofarabine	1.72E-03	0.95	1.10	2.07	1.40	1.23	1.17	1.86	2.00	2.39	2.09
M+Na-2H	1.26	319.07	C16H14N2O4	Amlexanox	6.13E-09	1.63		6.24	1.11	0.64		1.33		3.37	1.04
2M+H	3.87	265.11	C4H8N2O3	D-Asparagine	9.46E-05	0.98		9.61	1.32	0.84		1.86		2.11	
M+FA-H	1.26	352.00	C11H10BrN5O	2-oxobrimonidine	4.59E-06	1.01		4.88	1.31	1.18		1.82		3.35	1.02
M+Na	0.91	333.24	C19H34O3	(R)-2-Hydroxysterculic acid	4.09E-07	2.78		12.63	1.19	0.67		0.45		1.77	
M-H	5.95	880.18	C27H46N7O18P3S	(S)-Hydroxyhexanoyl-CoA	7.05E-04	2.58	1.37	1.29		0.69	1.29	0.33	1.36	0.58	
M+H-H2O, M+H	5.95	487.16	C18H32O16	3-beta-Cellobiosylglucose	1.77E-13	18.36	1.05	9.67		0.95		0.42		1.39	
M-H	1.02	630.42	C33H62NO8P	PE(14:1(9Z)/14:1(9Z))	1.81E-05	1.66	1.41	4.63	2.86	0.55	2.28	0.48	1.74	1.36	
M+Na-2H	0.95	695.51	C37H75N2O6P	SM(d18:1/14:0)	3.36E-05	1.70	1.06	5.78	2.11	0.70	1.19	0.44	1.24	1.26	
M+Na-2H	0.73	471.35	C28H50O4	6-Deoxocastasterone	5.26E-13	1.60		3.92	1.15	0.52		0.41		1.28	
M+H	0.99	523.11	C23H22O14	Spinatoside	1.01E-03	1.48		6.45	1.08	0.53		0.35		1.57	
2M+FA-H	0.95	757.54	C24H36O2	Tetracosahexaenoic acid, n-3	1.69E-04	1.44		3.16	1.51	0.90		0.38	1.33	1.15	
M+Na-2H	1.75	493.29	C29H44O5	(23S,24S)-17,23-Epoxy-24,29-dihydroxy-27-norlanost-8-ene-3,15-dione	2.70E-03	5.85	1.37	5.12		0.54		0.26		2.04	1.04

RT:Rentation Time

VIP:variable importance in projection

Supplementary Table S3 S-nitrosylated proteins in the seeds during controlled deterioration.

Protein name	Accession number	Mol mass (Mr) /Cal pI	Organism	Identified peptides			Cited as S-nitrosylated#	Ageing time		
				Sequence*	Score*	Sequence coverage (%)		0d	2d	5d
**General metabolism**										
Fructose-bisphosphate aldolase	W9QQH1	38418.31 /6.49	Morus notabilis	K.GILAADESTG TIGK.R	94.97+3	15.92%	Murray, C. I., et al. (2012); Kohr, M. J., et al. (2011); Abat, J. K., et al. (2008); Lindermayr et al.,(2005).	√	√	√
Triosephosphate isomerase	M5X2A0	27278.99 /6	Prunus persica	K.VASPAQAQE VHFELR.K	95.15+4	21.65%	Shi, Q., et al. (2008); Murray, C. I., et al. (2012); Kohr, M. J., et al. (2011); Abat, J. K., et al. (2008); Zhao et al., (2016).	√	√	√
Glyceraldehyde-3-phosphate dehydrogenase	M5WY86	36567.5 /7.68	Prunus persica	K.DAPM*FVVG VNEK.D	66.8+3	8.01%	Lin, A., et al. (2012); Rodriguez-Pascual et al. (2008); Hara et al. (2005); Shi, Q., et al. (2008); Murray, C. I., et al. (2012); Kohr, M. J., et al. (2011); Forrester, M. T. (2009) ; Zhao et al., (2016).	√	√	√
Malate dehydrogenase	W9RPQ1	35591.58 /6.11	Morus notabilis	R.LNVQVSDVK. N	64.59+2	9.01%	Sun, J., et al. (2007); Kohr, M. J., et al. (2011).	√	√	√
ATP synthase subunit beta	F8TR99	44601.58 /5.15	Ulmus macrocarpa	R.DVNEQDVLL FIDNIFR.F	5.15+1	8.47%		√	√	√
Isoflavone reductase related protein	O81355	33822.15 /6.02	Pyrus communis	R.FFPSEFGNDV DR.V	35.68+1	7.14%		√	√	√
3-ketoacyl-CoA thiolase 2	W9RJR4	48666.32 /8.59	Morus notabilis	K.DTHADDLLA PVLK.A	49.41+3	6.68%	Foster, M. W., et al. (2009).	√	√	√
Nucleoside diphosphate kinase	W9SL33	16332.51 /6.84	Morus notabilis	K.IIGATNPAESA PGTIR.G	69.26+2	25.68%	Foster, M. W., et al. (2009).	√	√	√
Phosphoglycerate kinase	M5WN82	50384.48 /8.42	Prunus persica	K.GVTTIIGGGD SVAAVEK.V	44.37+1	5.63%	Abat, J. K., et al. (2008).		√	√
Succinyl-CoA ligase [ADP-forming] subunit beta	W9R0G6	45263.87 /6.07	Morus notabilis	K.GGTSIEDLAE K.F	42.63+1	4.27%	Kohr, M. J., et al. (2011).		√	√
ATP synthase subunit alpha (Fragment)	Q5IBJ3	45497.78 /7.78	Ficus pumila	R.VVDALGVPID GR.G	49.75+2	2.83%	Qu, Z., et al. (2014); Martínez-Ruiz, A. and S. Lamas (2007); Kohr, M. J., et al. (2011); Abat, J. K., et al. (2008); Palmieri et al., (2010).		√	√

2,3-bisphosphoglycerate-independent phosphoglycerate mutase	O24246	53393.91<br>/5.38	Prunus dulcis	R.GWDAQVLGE<br>APHK.F	36.16+1	5.12%				√
6-phosphogluconate dehydrogenase, decarboxylating	M5WX92	53690.85<br>/6.24	Prunus persica	R.LPANLVQAQ<br>R.D	51.23+1	3.69%	Foster, M. W., et al. (2009).			√
Enoyl-ACP reductase	B8K204	41407.78<br>/8.72	Malus domestica	R.IIPGYGGGMS<br>SAK.A	54.06+1	5.36%				√
UDP-arabinopyranose mutase 3	W9QNZ0	41054.87<br>/5.77	Morus notabilis	K.DELDIVIPTIR.<br>N	44.88+3	9.39%				√
Reversibly glycosylated polypeptide	W9QXM6	41479.34<br>/6.26	Morus notabilis	K.DELDIVIPTIR.<br>N	44.88+3	9.32%				√
Alpha-1,4-glucan-protein synthase [UDP-forming] 2	W9RHT6	40469.09<br>/5.65	Morus notabilis	K.YIYTIDDDCF<br>VAK.N	54.91+3	11.20%				√
Putative cytochrome c	W9RNP9	12316.89<br>/9.46	Morus notabilis	K.NMAVNWEE<br>K.T	26.04+1	17.70%				√
Adenylate kinase B	W9RSI3	26430.26<br>/7.65	Morus notabilis	K.GELVSDDLV<br>VGIIDEAMK.K	63.85+1	12.03%	Shi, Q., et al. (2008).	√		
Transketolase	W9RGS5	80304.82<br>/6.31	Morus notabilis	R.FLAIDAVEK.A	21.69+1	2.41%	Abat, J. K., et al. (2008).	√		
4-hydroxy-4-methyl-2-oxoglutarate aldolase	W9QJ00	17377.85<br>/5.53	Morus notabilis	K.VFEDNVLVR.<br>E	46.25+1	13.25%		√	√	
Aldehyde dehydrogenase family 7 member A1	W9QSH7	65293.91<br>/9.05	Morus notabilis	R.QIGDAFR.A	41.09+1	2.50%	Qu, Z., et al. (2014); Forrester, M. T. (2009).	√	√	
Putative mannitol dehydrogenase	W9R066	39452.47<br>/6.86	Morus notabilis	K.ETQEM*IDFA<br>AK.H	60.96+1	5.77%			√	
V-type proton ATPase subunit E	W9RQ18	26207.95<br>/7.03	Morus notabilis	K.VLQAQDDVV<br>NSM*K.E	29.36+1	9.13%	Foster, M. W., et al. (2009).		√	
hosphoglucomutase	W9RGV2	63511.24<br>/5.65	Morus notabilis	R.YDYENVDAG<br>AAK.E	34.04+2	5.67%	Qu, Z., et al. (2014); Foster, M. W., et al. (2009).	√		√
Pyrophosphate--fructose 6-phosphate 1-phosphotransferase	W9QWQ<br>4	69526.18<br>/7.25	Morus notabilis	K.NQGGYDLLG<br>R.T	45.42+1	3.65%		√		√

**Redox related**										
Thioredoxin H-type 1	W9S3Z3	19227.16 /5.29	Morus notabilis	K.KTPEVIFLK.V	30.26+1	5.17%	Lin, A., et al. (2012); Martínez-Ruiz, A. and S. Lamas (2007)		√	√
2-Cys peroxiredoxin BAS1-like protein	W9S0A5	28967.77 /7.7	Morus notabilis	R.GLFIIDK.E	30.86+1	5.70%				√
1-Cys peroxiredoxin	W9RAN6	24457.55 /6.17	Morus notabilis	R.NMDEVLR.V	34.66+1	7.73%	Anand, P. and J. S. Stamler (2012)	√	√	
**Protein regulation**										
T-complex protein 1 subunit gamma	W9RFH2	151281.8 /5.64	Morus notabilis	R.IDDIVSGIK.K	44.05+1	1.25%	Qu, Z., et al. (2014); Forrester, M. T. (2009).	√	√	√
RuBisCO large subunit-binding protein subunit alpha	W9SAI5	61923.1 /5.26	Morus notabilis	K.TNDSAGDGTTTASVLAR.E	51.58+7	14.53%	Abat, J. K., et al. (2008).	√	√	√
Peptidyl-prolyl cis-trans isomerase	W9RP45	22012.03 /9.15	Morus notabilis	K.FADENFK.L	36.81+2	12.75%	Murray, C. I., et al. (2012); Kohr, M. J., et al. (2011); Zhao et al., (2016).	√	√	√
Proteasome subunit alpha type	M5WHF6	25570.79 /5.73	Prunus persica	K.NIEIGIIGTDK.K	52.33+2	14.47%	Qu, Z., et al. (2014); Foster, M. W., et al. (2009).	√	√	√
Chaperone protein	W9RSC3	101469.6 /5.81	Morus notabilis	K.TAVVEGLAQR.I	28.25+1	2.52%		√	√	√
Chaperonin CPN60-2	W9RE69	40809.06 /9.31	Morus notabilis	K.TLYNELEVVEGMK.L	83.29+3	10.32%		√	√	√
Elongation factor 1-alpha	Q9ZRP9	49480.7 /9.15	Malus pumila	R.QTVAVGVIK.S	62.34+5	11.86%	Forrester, M. T. (2009); Abat, J. K., et al. (2008); Lindermayr et al.,(2005).	√	√	√
Elongation factor 2	W9R0F3	98439.45 /5.69	Morus notabilis	K.EGALAEENMR.G	68+5	6.02%	Murray, C. I., et al. (2012); Abat, J. K., et al. (2008); Lindermayr et al.,(2005).	√	√	√
Eukaryotic translation initiation factor eIF5A	Q2L998	17132.07 /5.3	Rosa chinensis	R.LPTDDALLTQLK.D	55.38+1	14.01%	Qu, Z., et al. (2014); Murray, C. I., et al. (2012); Forrester, M. T. (2009); Abat, J. K., et al. (2008).	√	√	√
RuBisCO large subunit-binding protein subunit beta	W9QWD3	82849.64 /5.58	Morus notabilis	K.VVAAGANPVLITR.G	62.36+4	8.19%			√	√
Eukaryotic initiation factor 4A-8	W9QZE3	48038.6 /5.55	Morus notabilis	K.GLDVIQQAQSGTGK.T	45.55+2	7.33%			√	√
ribosomal protein S5	W9RQK6	22743.91 /9.76	Morus notabilis	K.TIAECLADELINAAK.G	56.31+2	15.69%			√	√

Ubiquitin-conjugating enzyme E2 35	W9RHZ4	17289.71 /6.16	Morus notabilis	K.WSPALQIR.T	41.13+2	16.99%	Qu, Z., et al. (2014); Foster, M. W., et al. (2009); Forrester, M. T. (2009).			√
Vacuolar-sorting receptor 1	W9RG58	69419.13 /5.4	Morus notabilis	R.YCAPDPEQDFSR.G	44.42+1	3.66%				√
40S ribosomal protein S28	W9SDZ8	7521.68 /11.16	Morus notabilis	R.EGDILTLLESER.E	66.09+4	40.00%	Murray, C. I., et al. (2012); Foster, M. W., et al. (2009).			√
20 kDa chaperonin	W9R4V1	26746.38 /9	Morus notabilis	K.TAGGLLLTEASK.E	59.65+1	8.63%				√
Nascent polypeptide-associated complex subunit alpha-like protein	W9S3Y1	22292.29 /4.37	Morus notabilis	K.SPTSDTYVIFGEAK.I	97.46+3	15.27%	Foster, M. W., et al. (2009)	√		
ADP-ribosylation factor 2	W9S0L5	21082.94 /6.43	Morus notabilis	R.ILMVGLDAAGK.T	36.44+1	11.35%	Foster, M. W., et al. (2009).	√		
Translationally-controlled tumor protein homolog	O03992	19038.23 /4.37	Fragaria ananassa	K.VVDIVDTFR.L	54.7+1	9.41%		√		
Elongation factor 1-delta	W9S9G6	95113.86 /8.63	Morus notabilis	K.APSAEYVNVSR.W	40.33+1	3.19%	Qu, Z., et al. (2014); Kohr, M. J., et al. (2011); Forrester, M. T. (2009).	√		
T-complex protein 1 subunit delta	D9ZJD4	57731.47 /7.08	Malus domestica	R.GSNQLVLDEAER.S	37.52+1	3.93%	Murray, C. I., et al. (2012).	√	√	
Oligopeptidase A	W9RQH4	91291.79 /6.03	Morus notabilis	K.FEENVLDATK.K	41.05+1	2.10%			√	
Chaperonin 60 subunit beta 2	W9RVQ7	64048.56 /5.55	Morus notabilis	K.VVAAGANPVLITR.G	78.11+7	14.76%		√		√
Protein disulfide-isomerase	W9R743	56240.96 /4.92	Morus notabilis	K.QSGPASVEIK.S	66.05+1	3.98%	Qu, Z., et al. (2014); Murray, C. I., et al. (2012); Anand, P. and J. S. Stamler (2012).	√		√
40S ribosomal protein S20-2	W9RZK8	13732.88 /9.65	Morus notabilis	K.SPCGEGTNTWDR.F	78.10+2	19.67%		√		√
**Stress related**										
14-3-3 protein 4	W9RXJ7	81368.36 /5.89	Morus notabilis	K.SAQDIALAELAPTHPIR.L	48.9+5	6.63%	Foster, M. W., et al. (2009).	√	√	√
14-3-3-like protein GF14 kappa	W9S4K1	29014.54 /4.98	Morus notabilis	K.DSTLIM*QLLR.D	27.15+3	9.20%		√	√	√
Heat shock protein 60 (Fragment)	Q8H6U4	57762.96 /5.26	Prunus dulcis	K.TLYNELEVVEGMK.L	83.29+5	10.09%	Sun, J., et al. (2007); Qu, Z., et al. (2014); Kohr, M. J., et al. (2011).	√	√	√

Stromal 70 kDa heat shock-related protein	W9R0D5	75385.25/5.3	Morus notabilis	K.DIDEVILVGGSTR.I	67.5+3	6.51%		√	√	√
Heat shock 70 kDa protein 4	W9R8D4	70996.66/5.09	Morus notabilis	R.TTPSYVAFTDTER.L	57.28+12	20.99%	Martínez-Ruiz, A. and S. Lamas (2007); Kohr, M. J., et al. (2011); Huang, B., et al. (2012).	√	√	√
ER-binding protein	A9UKE0	73570.37/5.14	Malus pumila	R.QIDEIVLVGGSTR.I	65.59+11	19.49%		√	√	√
Endoplasmin-like protein	W9SBY5	114005.1/5.58	Morus notabilis	R.ELISNASDAIDK.I	75.21+2	2.49%		√	√	√
Luminal-binding protein 5	W9RWI9	73496.4/5.04	Morus notabilis	R.FEELNNDLFR.K	64.39+12	20.54%		√	√	√
Putative dnaK-type molecular chaperone hsc70.1 (Fragment)	A7Y7I0	20124.47/5.07	Prunus dulcis	K.ELESICNPIIAK.M	51.63+3	25.70%		√	√	√
Heat shock protein STI	W9R4M6	64854.34/6.14	Morus notabilis	K.ALETYQEGLK.H	39.57+1	3.31%			√	√
Calmodulin 1	A7LAX1	16847.5/4.11	Morus nigra	K.DQNGFISAAELR.H	64.59+1	13.42%			√	√
Leucine aminopeptidase 3	W9RX96	60314.29/6.02	Morus notabilis	K.IAATYSDVLSAK.I	53.63+2	6.04%			√	√
GTP-binding nuclear protein Ran-3	W9RXL6	34637.82/5.19	Morus notabilis	K.LVIVGDGGTGK.T	51.77+2	9.60%			√	√
Proliferation-associated protein 2G4	W9R290	46999.23/5.62	Morus notabilis	K.ELDLTSPEVVTK.Y	50.36+1	4.48%			√	√
Temperature-induced lipocalin	Q38JC4	21462.96/5.6	Prunus armeniaca	R.LDDEIYNQLVQR.A	71.88+2	11.35%				√
DnaJ-like protein	W9QJ41	48873.58/5.74	Morus notabilis	R.EIYDQYGEDALK.E	51.33+1	5.91%				√
Proliferating cell nuclear antigen	W9RPL7	29167.09/4.67	Morus notabilis	R.MPSAEFAR.I	35.77+1	6.08%	Qu, Z., et al. (2014); Murray, C. I., et al. (2012); Forrester, M. T. (2009).	√		
Heat shock 70 kDa protein 15	W9R6E1	94664.25/5.16	Morus notabilis	K.LQEVEDWLYEDGEDETK.G	49.97+1	3.02%		√		
Heat shock cognate protein 80	W9RXY8	83453.58/4.82	Morus notabilis	R.ELISNASDAIDK.I	75.21+6	10.25%		√		

Heat shock protein 17.8	A0MWF1	17886.13/8.83	Rosa chinensis	R.VLQISGER.N	41.62+1	9.74%			√	
Cysteine proteinase inhibitor 12	W9RNY7	26824.03/6.56	Morus notabilis	K.ENALLEFAR.V	27.73+1	6.28%			√	
Calreticulin	Q9XF98	48415.67/4.4	Prunus armeniaca	K.GIQTSEDYR.F	42.66+1	3.56%	Murray, C. I., et al. (2012)		√	
Heat shock 70 kDa protein	W9R5Z5	71197.77/5.07	Morus notabilis	R.TTPSYVAFTDSER.L	79.89+11	24.12%		√		√
**Other**										
Cell division cycle protein 48-like protein	W9RIC2	96188.29/5.17	Morus notabilis	R.EIDIGVPDEVGR.L	58.03+9	11.07%		√	√	√
Beta-hexosaminidase	M5X5L2	61296.81/6.41	Prunus persica	R.GLLLDTSR.H	34.48+1	2.95%				√
Actin 1	E7D7Z5	41695.33/5.31	Morus alba	K.AGFAGDDAPR.A	66.09+4	18.04%	Zhao et al. (2014); Martínez-Ruiz, A. and S. Lamas (2007).	√	√	
Actin 3 (Fragment)	E7D7Z4	35519.31/5.46	Morus alba	K.AGFAGDDAPR.A	66.09+4	19.20%		√	√	
Putative glycine-rich RNA-binding protein 1	E5L932	16187.03/6.36	Malus hupehensis	R.DAIEGM*NGQNLDGR.N	60.24+1	14.63%			√	

*The best-matching peptide identifying the protein and the score is given. If there were further peptides found, the number of the peptides is given as an additional number.

√ S-nitrosylated proteins during different controlled deterioration time.

[#] References of S-nitrosylated proteins found in previous studies.
