# Supplementary material for: Nitric oxide alleviates cell death through protein S-nitrosylation and transcriptional regulation during the ageing of elm seeds
Source: J Exp Bot. 2018 Jul 25;69(21):5141–55. doi: 10.1093/jxb/ery270 (PMC6184755; doi:10.1093/jxb/ery270)
Supplement: Supplementaty Figures S1-S4 [file ery270_suppl_supplementaty_figures-s1-s4.pdf]

Figure S1

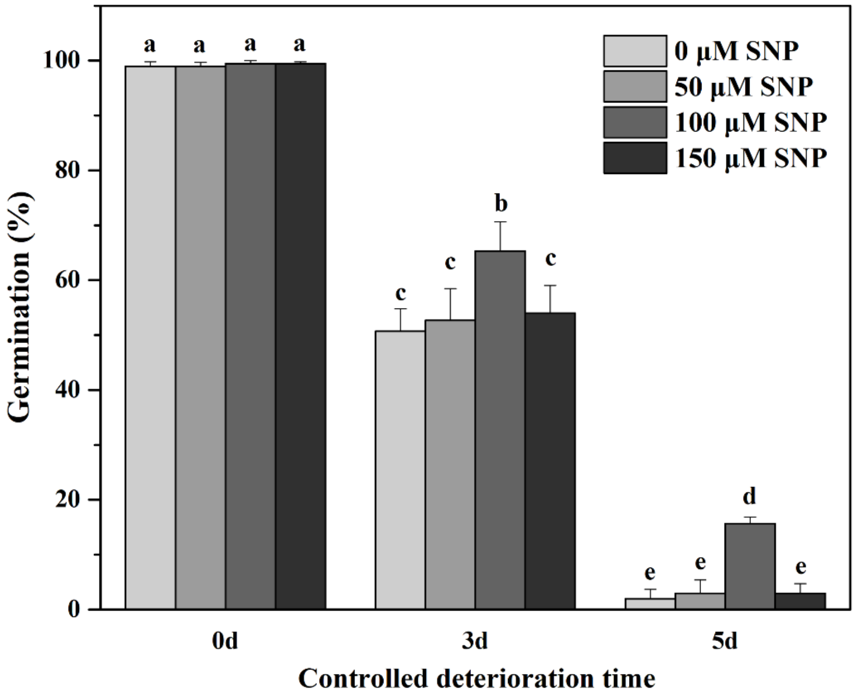

Supplementary Figure S1 Effect of the different concentrations of SNP on seeds subjected to CDT for 0 day, 3 days and 5 days. Seeds were treated with either water vapour or different concentrations of SNP vapours, and the percentage of germination was calculated after seven days of imbibition. Data were presented as the mean±standard derivation of three replicates. Different superscript letters indicate significant differences at *P*<0.05.

Figure S2

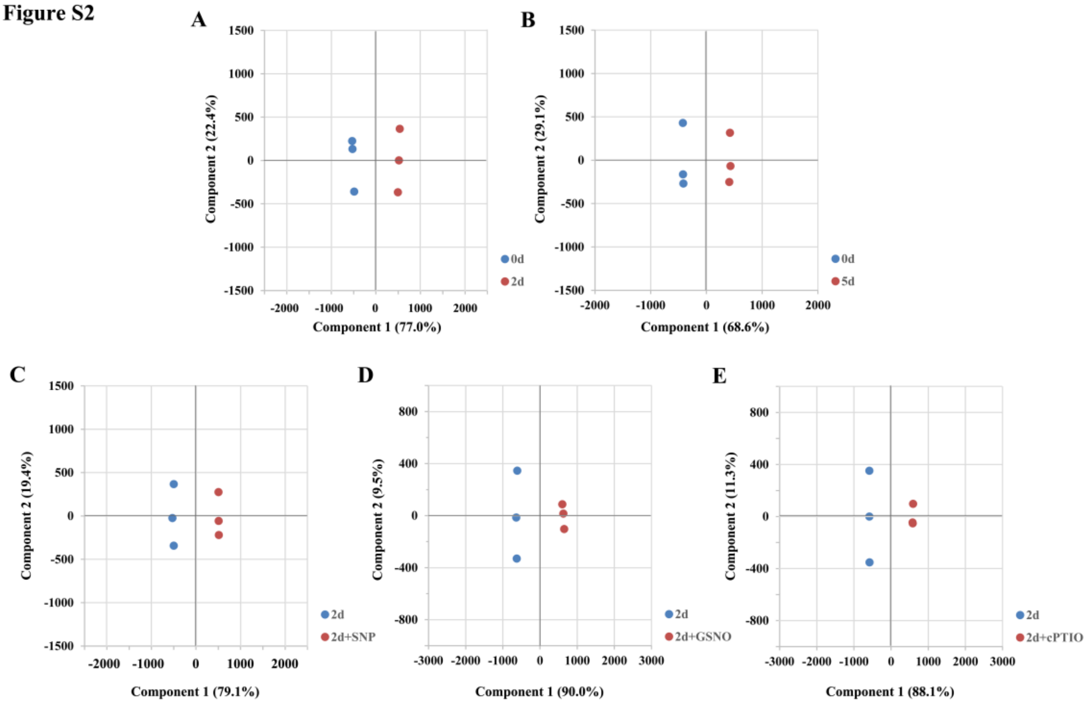

Supplementary Figure S2 OPLS-DA score plots derived from the UPLC-Q-TOF MS spectra following CDT for 0 d and 2 d (A), CDT for 0 d and 5 d (B), CDT for 2 d and 2 d+SNP (C), CDT for 2 d and 2 d+GSNO (D), CDT for 2 d and 2 d+c-PTIO (E).

Figure S3

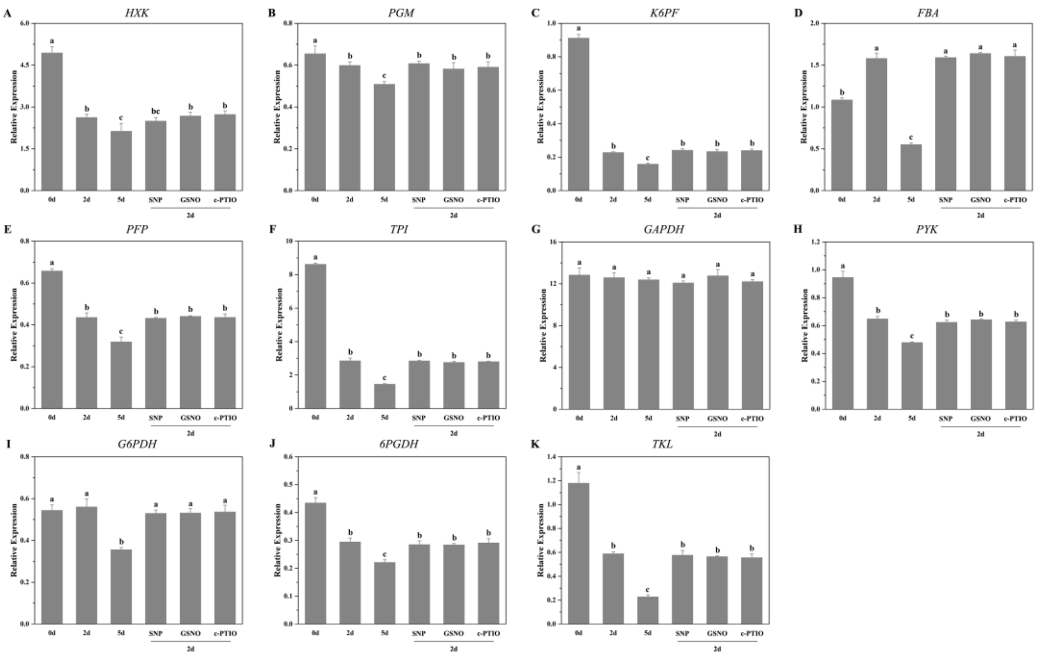

Supplementary Figure S3 The expression pattern of genes in carbohydrate metabolism with different treatments using qRT-PCR. $P<0.05$. *HXK*, hexokinase; *PGM*, phosphoglucomutase; *K6PF*, 6-phosphofructokinase; *FBA*, fructose bisphosphate aldolase; *PFP*, pyrophosphate-fructose 6-phosphate 1-phosphotransferase; *TPI*, triosephosphate isomerase; *GAPDH*, glyceraldehyde-3-phosphate dehydrogenase; *PYK*, pyruvate kinase; *G6PDH*, glucose-6-phosphate 1-dehydrogenase; *6PGDH*, 6-phosphogluconate dehydrogenase; *TKL*, transketolase.

Figure S4

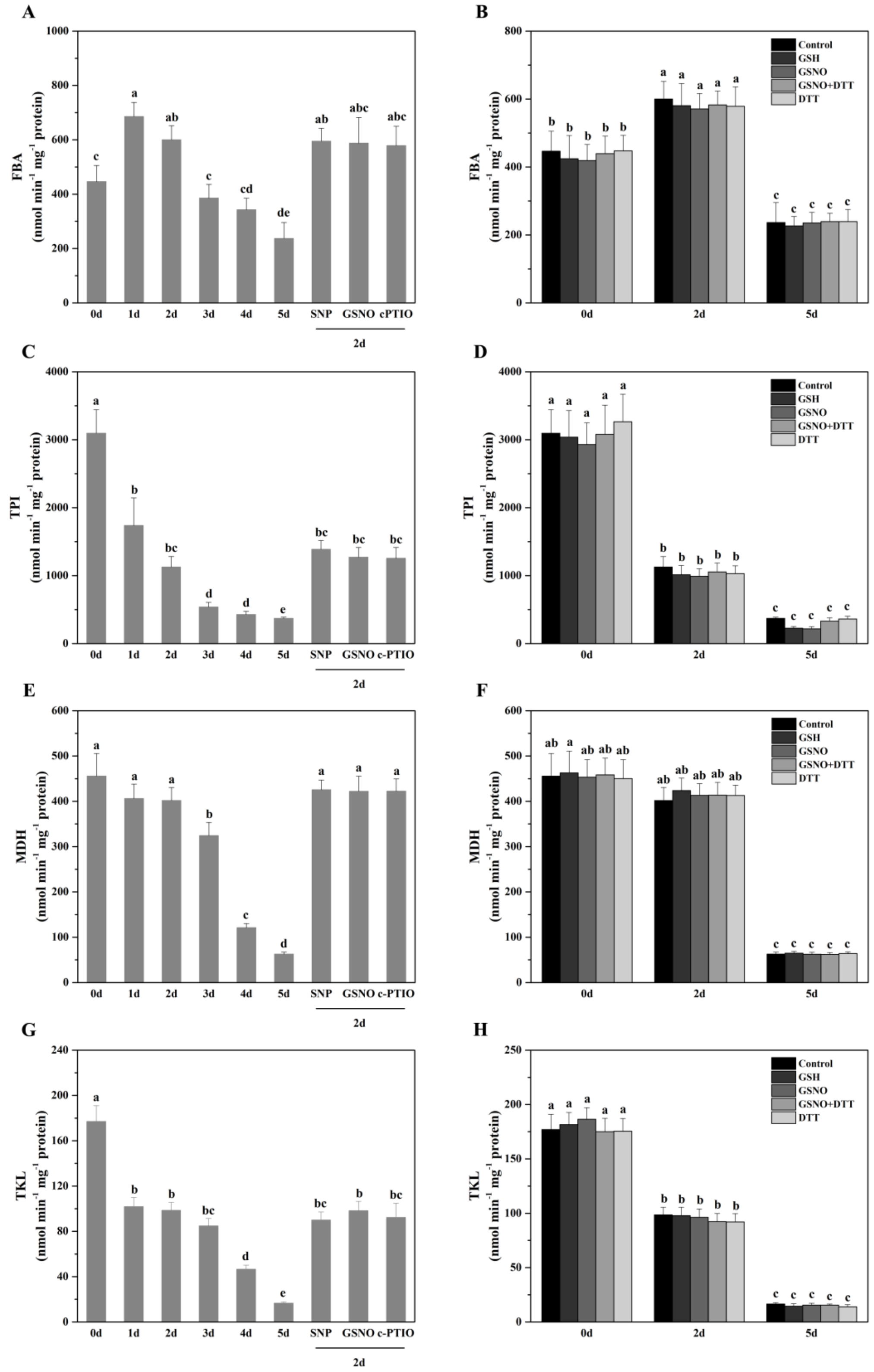

Supplementary Figure S4 Change of enzyme activities in four S-nitrosylated proteins during seed ageing.

(A, C, E, G) Effect of the CDT and NO on the enzyme activities of FBA (A), MDH (C), TKL (E)

and TPI (G). Seeds were subjected to CDT for different times or pre-treated with 100 μM SNP, 100 μM GSNO or 50 μM c-PTIO and subjected to CDT for two days. The crude extracts were used for the activity assays. Data are the mean±standard derivation of three replicates. Different superscript letters indicate significant differences at $P<0.05$. FBA, fructose-bisphosphate aldolase; MDH, malate dehydrogenase; TKL, transketolase; TPI, triosephosphate isomerase.

(B, D, F, H) Effects of the GSH and GSNO treatments on the enzymatic activity of FBA (B), MDH (D), TKL (F) and TPI (H) in ageing seeds. Crude extracts of aged elm seeds were treated with 250 μM GSH, 100 μM GSNO, 100 μM GSNO+10 mM DTT and 10 mM DTT and used for activity assays.
